# Nerve transfer for restoration of lower motor neuron-lesioned bladder, urethral and anal sphincter function. Part 4: Effectiveness of the motor reinnervation

**DOI:** 10.1152/ajpregu.00248.2023

**Published:** 2024-03-18

**Authors:** Ekta Tiwari, Danielle S. Porreca, Alan S. Braverman, Lewis Holt-Bright, Nagat A. Frara, Justin M. Brown, Benjamin R. Johnston, Stanley F. Bazarek, Brendan A. Hilliard, Michael Mazzei, Michel A. Pontari, Daohai Yu, Michael R. Ruggieri, Mary F. Barbe

**Affiliations:** ^1^School of Engineering, Brown University, Providence, Rhode Island, United States; ^2^Center of Translational Research, Lewis Katz School of Medicine at Temple University, Philadelphia, Pennsylvania, United States; ^3^Medical Doctor Program, Thomas Jefferson Research, Thomas Jefferson University Hospital, Philadelphia, Pennsylvania, United States; ^4^Aging and Cardiovascular Discovery Center, Lewis Katz School of Medicine at Temple University, Philadelphia, Pennsylvania, United States; ^5^Department of Neurosurgery, Massachusetts General Hospital, Boston, Massachusetts, United States; ^6^Department of Neurosurgery, Brigham and Women’s Hospital, Boston, Massachusetts, United States; ^7^Department of Neurological Surgery, Brigham and Women’s Hospital, Boston, Massachusetts, United States; ^8^Department of Trauma Surgery and General Surgery, LeHigh Valley Health Network, Allentown, Pennsylvania, United States; ^9^Department of Urology, Lewis Katz School of Medicine, Temple University Health System, Philadelphia, Pennsylvania, United States; ^10^Center for Biostatistics and Epidemiology, Department of Biomedical Education and Data Science, Lewis Katz School of Medicine at Temple University, Philadelphia, Pennsylvania, United States

**Keywords:** bladder, detrusor pressure, functional electrical stimulation, motor neuron, retrograde dye

## Abstract

In pilot work, we showed that somatic nerve transfers can restore motor function in long-term decentralized dogs. We continue to explore the effectiveness of motor reinnervation in 30 female dogs. After anesthesia, 12 underwent bilateral transection of coccygeal and sacral (S) spinal roots, dorsal roots of lumbar (L)7, and hypogastric nerves. Twelve months postdecentralization, eight underwent transfer of obturator nerve branches to pelvic nerve vesical branches, and sciatic nerve branches to pudendal nerves, followed by 10 mo recovery (ObNT-ScNT Reinn). The remaining four were euthanized 18 mo postdecentralization (Decentralized). Results were compared with 18 Controls. Squat-and-void postures were tracked during awake cystometry. None showed squat-and-void postures during the decentralization phase. Seven of eight ObNT-ScNT Reinn began showing such postures by 6 mo postreinnervation; one showed a return of defecation postures. Retrograde dyes were injected into the bladder and urethra 3 wk before euthanasia, at which point, roots and transferred nerves were electrically stimulated to evaluate motor function. Upon L2-L6 root stimulation, five of eight ObNT-ScNT Reinn showed elevated detrusor pressure and four showed elevated urethral pressure, compared with L7-S3 root stimulation. After stimulation of sciatic-to-pudendal transferred nerves, three of eight ObNT-ScNT Reinn showed elevated urethral pressure; all showed elevated anal sphincter pressure. Retrogradely labeled neurons were observed in L2-L6 ventral horns (in laminae VI, VIII, and IX) of ObNT-ScNT Reinn versus Controls in which labeled neurons were observed in L7-S3 ventral horns (in lamina VII). This data supports the use of nerve transfer techniques for the restoration of bladder function.

**NEW & NOTEWORTHY** This data supports the use of nerve transfer techniques for the restoration of bladder function.

## INTRODUCTION

Normal bladder function (storage and emptying) following spinal cord injuries (SCIs) and lower spinal root injuries is difficult to restore as both the bladder and urethral sphincters contribute to aberrant reflexes. Detrusor hyperreflexia, a condition where uninhibited bladder contractions limit bladder storage, whereas detrusor areflexia, a condition that causes failure in the voluntary emptying bladder is seen in patients with SCI and with other upper motor neuron pathologies (1). In addition, in a retrospective study of 407 patients with pelvic fractures in which the sacrum is fractured, 46% had persistent neurological damage to lumbar level 5 to sacral level 5 (L5-S5) roots that pass through the sacrum, with lower extremity dysfunction and urinary and anal incontinence (2). Sacral roots and nerves are also affected by the removal of sacral chordomas (3, 4). These lower motor neuron abnormalities cause serious urological complications and damage to the lower urinary tract (LUT), and ultimately reduce quality of life (5–8). Therefore, restoration of bladder function, whether the result of upper or lower motor neuron injuries, is consistently identified as a top recovery priority for people with SCI (9).

Several studies have discussed or shown means to treat or manage lower motor neuron bladder dysfunction via nonpharmacological interventions, pharmacological interventions, surgical interventions, or implantable devices (1, 10–14). However, despite all these efforts and advances in managing or treating bladder dysfunction, neurogenic lower urinary tract dysfunction remains one of the most frequently reported health issues for individuals with spinal cord lesions in their lower lumbar and sacral regions (5, 8, 15, 16). Therefore, the search continues for improved treatments that would restore lower urinary tract function.

In the past, our laboratory has successfully demonstrated functional motor reinnervation of the bladder in lower motor neuron lesioned canines by developing new surgical approaches. Improved bladder function was demonstrated after: *1*) immediate end-on-end repair after transection of sacral roots inducing bladder contraction (17); *2*) immediate transfer of coccygeal roots in the lumbosacral spinal column to severed bladder sacral roots (18); *3*) immediate transfer of the genitofemoral nerve (a mixed sensory and motor somatic donor nerve) to the vesical branch of the pelvic nerve in the lower abdomen (19); and even *4*) genitofemoral-to-pelvic nerve transfer at 1 and 3 mo after denervation to more closely mimic the possible clinical scenario (19). We have shown that bladder reinnervation using immediate transfer of a primarily motor nerve (femoral nerve) transfer to the pelvic nerve is functionally superior to the genitofemoral nerve for return of motor function (20). In each of these studies, bladder reinnervation was confirmed using functional electrical stimulation evoked bladder contraction and emptying, and retrograde labeling of spinal cord neurons following dye injections into the bladder wall. We have also demonstrated improved urethral and anal sphincter function in three dogs after the immediate transfer of motor branches of the femoral nerve to the pudendal nerve after sacral root transection (21). However, we still need to demonstrate motor reinnervation of the bladder, urethra, and anal sphincter after long-term decentralization, and reinnervation to determine the potential of surgical rerouting for restoration of voiding function and fecal and urinary continence after lower lumbar and sacral neural injury.

To meet this challenge, we first published an interim pilot study with a small number of animals that suggested that bilateral somatic nerve transfer to pelvic and pudendal nerves (specifically, transfer of a portion of the obturator nerve to the vesical branch of the pelvic nerve, and transfer of a redundant branch of the sciatic nerve to the pudendal nerve can potentially restore bladder sensation and motor function in long-term bladder, urethra and anal sphincter decentralized dogs (22). Three (of three) reinnervated animals showed recovery of squat-and-void postures in their home cage at 4–6 mo after a year-long decentralization period (defecation postures were not tracked in that study). One of these even showed voluntary voiding after awake bladder filling. We then expanded that study to include additional animals, using the same somatic nerve transfer strategy, in a series of three papers to date. In Parts 1 and 3, we used ex vivo muscle strip electrophysiological methods to examine the effects of long-term decentralization, with or without the nerve transfers, on the contributions of muscarinic, purinergic, and nicotinic receptors to bladder smooth muscle contractions using detrusor muscle strips (23, 24). In Part 2, we examined histological and axonal innervation changes in the bladder walls of these same animals, and if those changes correlated with peripheral nerve-evoked bladder contraction (25). We still seek to demonstrate motor reinnervation of bladder, as well as the urinary and anal sphincters, after long-term decentralization.

Thus, our objectives here were to assess the effectiveness of reinnervation in nerve-transferred animals after long-term denervation (∼18–22 mo) by examining micturition and defecation behaviors and functional electrical stimulation of spinal roots and spinal segments of L2-S3 and transferred nerves, and to examine retrograde labeling from the end organs to ventral horns of the spinal cord to further confirm the extent of motor reinnervation. Based on findings from our pilot study (22), we hypothesize that our nerve transfer strategies will improve bladder emptying and continence, although our histological assays of the peripheral coaptation site and bladder suggest that surgical- and denervation-related fibrosis may negatively affect the outcomes (25). Therefore, further investigations in this animal model are needed before these surgical procedures can be applied to human patients with lower motor neuron lesioned bladders and sphincters.

## MATERIALS AND METHODS

### Animals

Before onset, the protocol for this study was submitted to and approved by the Temple University Institutional Animal Care and Use Committee in accordance with the guidelines of the National Institute of Health for the Care and Use Laboratory Animals, United States Department of Agriculture, and Association for Assessment and Accreditation of Laboratory Animal Care (AAALAC). Upon approval, this study was then under the oversight of the Temple University Institutional Animal Care and Use Committee and in accordance with the guidelines of the National Institute of Health for the Care and Use Laboratory Animals, United States Department of Agriculture, and AAALAC.

Thirty female mixed-breed mongrel hound dogs were included in this study that were 6–8 mo of age and 20–25 kg body weight at experimental onset, from Covance Research Products, Inc., Lancaster County, PA or Marshall BioResources, North Rose, NY. Animals were housed in an AAALAC-accredited central animal facility and provided free access to food and water and maintained in a 12:12 h light-dark cycle in 22–24°C housing rooms. They were housed in large, connected pens, typically with 1 or 2 cage companions. Standard environmental enrichment and/or exercise were performed during group/single housing as per protocol.

### Original Design and Necessary Modifications of the Project Plan

**Figure 1.**
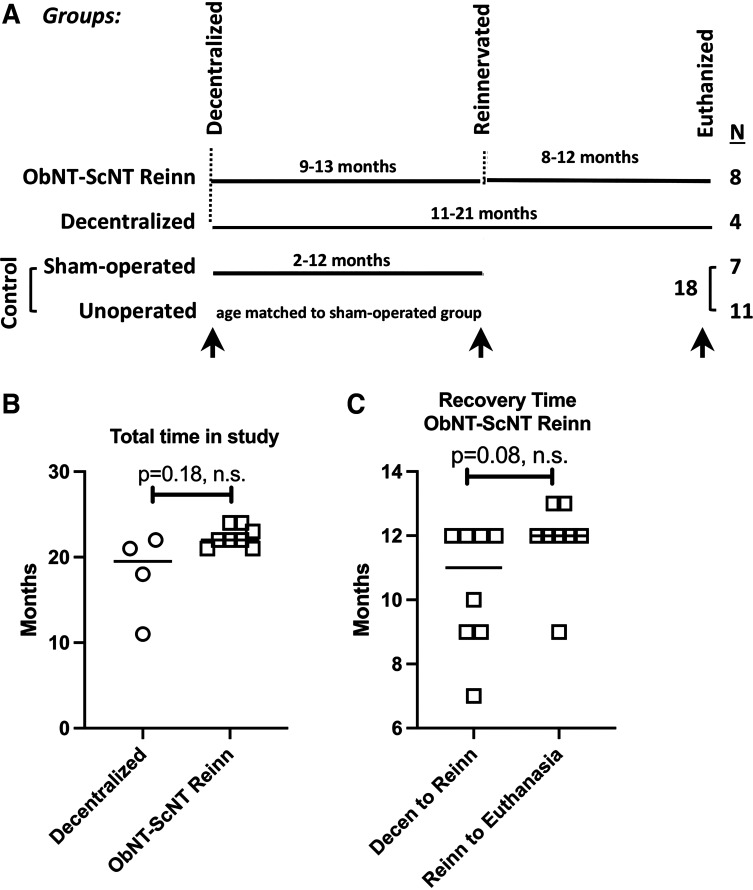
*A*: study design. There were three main groups and a total of 30 animals. *Group 1* were ObNT-ScNT Reinn animals (*n* = 8) that were decentralized [transection of dorsal root of lumbar (L) 7, dorsal and ventral roots of sacral (S) 1-3, and hypogastric nerves, each bilaterally] followed by an average 12 mo recovery (9–13 mo range); then, a portion of the obturator nerve was transferred to the pelvic nerve’s vesical branch, followed by an average recovery time of 10 mo (8–12 mo range). *Group 2* dogs were similarly decentralized as *group 1*, followed by an average 18 mo recovery (11–21 mo range; Decentralized; *n* = 4). *Group 3* dogs were Controls that included sham-operated (*n* = 7) and unoperated (*n* = 11) animals for *N* = 18 total. Arrows indicate the behavioral testing time points reported; *n* = number of animals per group. *B*: total time in study for Decentralized and ObNT-ScNT Reinn animals. *C*: recovery time for ObNT-ScNT Reinn animals showing months between decentralization and reinnervation and months between reinnervation and euthanasia. n.s. = not significant.

The original design and necessary modifications of the project plan are as described in Part 1 of this series (26). The current study is Part 4 of a series reports and results from 30 animals with three main groups (Fig. 1*A*). The ObNT-ScNT Reinn group consisted of eight dogs that were in the study a total of 22 mo (22 ± 0.4 mo, means ± SE, Fig. 1*B*). At study onset, these ObNT-ScNT Reinn animals functionally decentralized by bilaterally transecting the dorsal roots of L7, all spinal roots caudal to L7, and the hypogastric nerves, followed by a 9- to 13-mo recovery period (10.4 ± 0.7 mo, Fig. 1*C*), then reinnervation by transfer of the obturator nerve to the vesical branch of the pelvic nerve, as well as a branch of the sciatic nerve to the pudendal nerve, that was then followed by an additional 8–12 mo recovery (11.9 ± 0.4 mo, Fig. 1*C*). The Decentralized group consisted of four animals that underwent similar decentralization followed by an 11–21 mo recovery (18 ± 2.5 mo, Fig. 1*B*) but no reinnervation surgeries. Controls consist of 7 sham-operated and 11 unoperated animals (18 total; Fig. 1*A*).

There is some overlap with the pilot study (22) and Part 2 (25) of this series. Specifically, we included squat-and-void posture data from three ObNT-ScNT Reinn animals, one Decentralized animal, and eight Control animals (5 sham-operated and 3 sham-unoperated) from the small pilot study, and in vivo electrophysiology data for peripheral nerve-evoked bladder, urethra and anal sphincter contractility from 3 ObNT-ScNT Reinn animals from the pilot study (22). We also included the in vivo peripheral nerve-evoked bladder contractility graph from Part 2 of this series (25) for comparison purposes to new additional data.

Unique to this paper are the following: *1*) data from 18 additional animals, *2*) report of defecation postures; *3*) segmental spinal root/cord-evoked bladder, urethra, and anal sphincter contractility data from all animals from L2-S3 to more closely match reports that spinal cord input to the obturator nerve is nearer to L3-L6 (20, 27–29); *4*) peripheral nerve-evoked urethra and anal sphincter contractility data from the Decentralized and Control animals, and five additional ObNT-ScNT Reinn animals; *5*) retrograde dye labeling data in the spinal cord ventral horn segments from L2-S3 after dye injections into the bladder and urethra sphincter; *6*) Rexed laminar location of these labeled neurons in the spinal cord; and *7*) correlations between these various outcomes. Because of the inclusion of the urethral and anal sphincter data, we renamed the reinnervated group to ObNT-ScNT Reinn (different from prior studies in which we focused on the obturator nerve transfer to the pelvic nerve results, and thus named the reinnervated group as “ObNT-Reinn”).

### Decentralization of the Bladder and Nerve Transfer Surgeries

Surgical decentralization and nerve transfer procedures were as previously described (22, 25, 26). Briefly, dogs were sedated with propofol (6 mg/kg iv) for endotracheal intubation and then anesthetized using isoflurane (2–4% maximum alveolar concentration) with oxygen. Double-balloon catheters were placed in the urethra and bladder (30). All animals except for unoperated controls underwent laminectomy of L6-S3 vertebrae. L7 and S1-3 ventral roots were identified electrophysiologically, as previously described (17, 18). Decentralized and ObNT-ScNT Reinn animals underwent decentralization from pelvic end organs by bilateral extradural transection of dorsal roots of L7, bilateral extradural transection of dorsal and ventral roots of S1-3, and bilateral transection of hypogastric nerves within the abdomen. Dorsal root ganglia (L7-S3) were also excised in these animals, except for the four animals from the pilot study (three from the ObNT-ScNT Reinn group and one from the Decentralized group) (22). Sham-operated controls underwent lumbosacral laminectomy, nerve root identification using electrical stimulation without root transection, and abdominal laparotomy for identification of pelvic vesical nerve branches and hypogastric nerves.

For the nerve transfer surgeries, at 8–12 mo after decentralization, eight of the decentralized animals were reanesthetized and catheterized with balloon catheters, as described above. For reinnervation of the bladder detrusor muscle, obturator nerves were accessed abdominally, and divided longitudinally using a microscalpel; approximately 25% of the fascicles were transected, transferred, and sutured end-to-end to the transected vesical branch of the pelvic nerve, bilaterally, using described methods for identification of pelvic nerve branches (20), obturator nerve division (22), and end-on-end anastomosis (21). For reinnervation of external urethral and anal sphincters, a redundant branch of the sciatic nerve was identified in the posterior midthigh and then transferred cranially to branches of the pudendal nerve that induced urethral and anal sphincter contractions with intraoperative electrical stimulation (22) [the pudendal nerve and its branches were identified within Alcock’s canal (31)]. Axoguard nerve connectors (Axogen Corp, Alachua, FL) were used to maintain transferred nerve coaptation and to reinforce the coaptation site which was covered in Tisseel fibrin sealant (Baxter, Deerfield, IL) (26).

### Postoperative Care

Postoperative care procedures were as described previously (22, 26). Urine samples were collected, and urinalysis results were previously reported in Part 1 of this series (26).

### Observation of Squat-and-Void and Defecation Postural Behaviors

Squat-and-void postures and defecation postures were tracked pre- and postdecentralization, and postreinnervation by recording them for 24 h at monthly intervals in five of the sham-operated control, all four Decentralized, and all eight ObNT-ScNT Reinn animals. Postures/day are reported for three time points: presurgery, postdecentralization (recorded during the month immediately before the nerve transfer surgery in the reinnervated group and half-way to euthanasia for the other two groups), and final (a recording was made within the month before terminal surgery). These videos were assessed by observers not aware of the animals’ treatment allocation.

### Retrograde Dye Injections

Dogs were injected with retrograde labeling dyes three weeks before euthanasia, as previously described (30) using a telescope (Hopkins II 30″ Telescope, 2.9 mm × 30 cm and Tele Pack x LED, TP 100, Karlz Storz, Tuttlingen, Germany). Briefly, animals were sedated and anesthetized, and the bladder was cystoscoped. Dogs received injections of Fluoro-Gold (FG; 4–5% wt/vol in 0.9% saline solution, Fluorochrome, LLC, Denver, CO) into the detrusor muscle at four different sites lateral to each ureteral orifice, and True Blue (TB; 2% wt/vol in 74% dimethyl sulfoxide, Life Technologies Corporation, Grand Island, NY) into four different sites of the urethral sphincter. All dogs except 3 of 11 unoperated control received these dye injections. All dogs were allowed to recover from anesthesia before being returned to their home cages.

### In Vivo Functional Electrical Stimulation at 10 Mo after Reinnervation Surgery or 18 Mo Postdecentralization

An average 10-mo reinnervation recovery time was chosen based on data from the pilot study showing functional recovery of squat-and-void postures between 4 and 6 mo after obturator nerve transfer in 3 ObNT-ScNT Reinn animals (32). For this, before euthanasia, the animals were anesthetized and catheterized, as described for decentralization. Bladder, external urethral sphincter, rectal, and anal sphincter pressures were monitored throughout the surgeries, as described (18), as were vital signs. Three successive filling cystometrograms were obtained to determine bladder capacity, as previously described (33). Detrusor pressure was calculated by subtracting rectal pressure (as an estimate of intra-abdominal pressure) from bladder pressure (also termed intravesical pressure). After laminectomy, L1-S3 spinal ventral roots or spinal cord segments were systematically stimulated (0.5–10 mAmp, 20-Hz, and a pulse duration of 0.2 ms in a train of 4–7 s duration), bilaterally, using handheld monopolar or bipolar electrodes while recording the induced bladder, urethra or anal sphincter pressures. Evoked maximum detrusor pressure (MDP), maximum urethral sphincter pressure (MUSP), maximum anal sphincter pressure (MASP) data was recorded at a 10-Hz sampling rate and is reported as an average for each stimulated root/spinal cord segment, as well as after binning the data in L2-6 and L7-S3 groupings, since in dogs, L7-S3 roots are the primary segments innervating the bladder (30), and the obturator nerve primarily originates from L3-L6 spinal cord segments in a variable range as described earlier. We included L2 data in the binning data due to past findings from the laboratory that this segment also induces bladder contraction (32). In addition, right and left transferred nerves (obturator-to-pelvic and sciatic-to-pudendal) were identified and stimulated in ObNT-ScNT Reinn animals, as were right and left intact pelvic and pudendal nerves in Control and Decentralized animals (0.5–10 mAmp, 20-Hz, and a pulse duration of 0.2 ms in a train of 4–7 s duration). Changes in pressures were continuously recorded with external pressure transducers interfaced with the PowerLab multichannel data acquisition system and LabChart software (ADInstruments, Colorado Springs, CO). The strength of evoked contractions was derived from differences between the resting baseline pressure and the peak pressure obtained during continuous stimulation. The resulting MDP, MUSP, and MASP are reported in cmH_2_O.

### Euthanasia and Tissue Collection

After the above electrophysiological testing, all animals were euthanized with Euthasol (Virbac Corporation, Fort Worth, TX; 1 mL for each 4.5 kg of body weight iv). Thereafter, the spinal cord was collected from all animals from lumbar (L) level 2 caudally to the sacral (S) level 3, in a segmental manner. Each segment was fixed by immersion in 4% paraformaldehyde in 0.1 M phosphate buffer (pH 7.4) for 2 days. They were then equilibrated in 10% and then 30% sucrose in phosphate buffer for two days each, before being frozen in OCT compound (Optimal Cutting Temperature Compound, Sciagen, Fisher-Scientific, Hempstead, NH) for future cryosectioning. Samples were cryosectioned into 14 µm cross sections, with every fifth section mounted onto charged slides (Fisher Plus, Thermo Fisher Scientific, Inc., Waltham, MA). All sections were dried onto the slides overnight, washed in phosphate-buffered saline (PBS), and coverslipped with 80% glycerol in PBS as a mounting medium. Sections were quantitatively evaluated using fluorescence microscopes for the presence of retrogradely labeled cell bodies.

### Quantitative Analysis of Retrogradely Labeled Neuronal Cell Bodies

Three sections of each spinal cord segment, per dog and per vertebral level, from L2 to S3, were analyzed quantitatively and bilaterally for the number of Fluoro-Gold-labeled and True Blue retrogradely labeled neuronal cell bodies per area of the ventral horn assayed, using previously described methods (30, 34). Briefly, regions of interest were landmarked and circumscribed at ×40 magnification, using a ×4 objective, on live microscopic images [see Fig. 6 in Ruggieri et al. (21)]. All fields of the intermediate and ventral horns were sampled systemically at ×400 magnification (using a ×40 objective) in three nonadjacent sections per tissue. To avoid bias in estimating the number of neurons, only retrogradely labeled perikarya with a clearly visible nucleus were counted. The sum of labeled cells counted per retrograde dye was divided by the size of ventral horn region of the spinal cord segment assayed, to provide an estimate of the number of cells per squared millimeter per segment. In addition, the Rexed laminar location of the retrogradely labeled cells was recorded onto the cord maps to provide an estimate of the number of cells per squared millimeter per lamina and per segment [see Rexed lamina divisions used in Fig. 5 in Gomez et al. (20)].

### Statistical Analyses

Statistical analyses were performed using GraphPad Prism 10, as was the graphing (GraphPad Software, La Jolla, CA) for all but the retrograde dye labeling data, which were analyzed using SPSS (IBM SPSS Statistics, Armonk, NY). The level of significance was set at *P* < 0.05 for all analyses and adjusted *P* values are reported for the post hoc analysis results. All data are presented as means and 95% confidence intervals (CIs). The statistical inter-group comparisons were predefined before the data were collected.

Mixed-effects regression models were used for the analyses of repeated-measures of behavioral postures and spinal roots/cord evoked contractions, followed by multiple comparison post hoc tests, with results confirmed using SAS version 9.4 (SAS Institute, Inc., Cary, NC). Behavior postures (squat-and-void and defecations postures) were analyzed using two factors: surgical group and time point (presurgery, postdecentralization, and final), followed by Tukey–Kramer’s multiple comparison post hoc tests. Analyses of spinal roots/cord evoked MDP, MUSP, and MASP contractions were performed for individual segmental levels (L1-S3), or with the root/spinal cord segments combined (L2-6 vs. L7-S3), using two factors: surgical group and segment(s), followed by Fisher’s LSD multiple comparison post hoc tests. For data examining MDP, MUSP, and MASP responses after peripheral nerve stimulation, Kruskal–Wallis ANOVAs were used followed by Dunn’s multiple comparison post hoc tests. Control group statistics for peripheral nerve stimulation included only five sham-operated control animals since peripheral nerve stimulation was not performed in the other control animals. Analyses of the numbers of retrogradely labeled neurons in spinal cord ventral horns were first performed for each dye using repeated-measures mixed-effects models with two factors: surgical group and individual segment (from L1 to S3). Next, this same data was reanalyzed similarly after combining the data into L2-6 versus L7-S3 segmental groupings. In addition, this data was reanalyzed using a repeated-measures mixed-effects model and three factors: surgical group, segmental group (L2-6 vs. L7-S3), and lamina location of the labeled neurons (VII, VIII, and IX). These were followed by Tukey-Kramer multiple comparison post hoc tests. Because this set of analyses did not test a prespecified statistical null hypothesis, the results are exploratory, therefore, the calculated *P* values are interpreted as descriptive, not hypothesis testing (35). For succinctness sake, the statistical findings from the mixed-effects models are listed in Table A1 and post hoc multiple comparison results are depicted in the figures.

Correlations between outcomes were also performed (Pearson’s *r* correlations) to inform if the outcomes were linked and if the responsiveness of one outcome was linked to responsiveness to another outcome. For this, electrical stimulation responses of L2-L6 were averaged before use in the correlation assay, as were L7-S3 spinal root responses. Similarly, retrograde dyes result for L2-L6 ventral horn segments were averaged, as were L7-S3 dye results.

## RESULTS

### Observations following Surgery

The incidence of culture-confirmed bacteriuria and antibiotic treatment was described in Part 1 of this series (26). None of the reinnervated dogs lost ambulation abilities.

### Reinnervation Recovered Squat-and-Void Postures yet Not Defecation Postures

**Figure 2.**
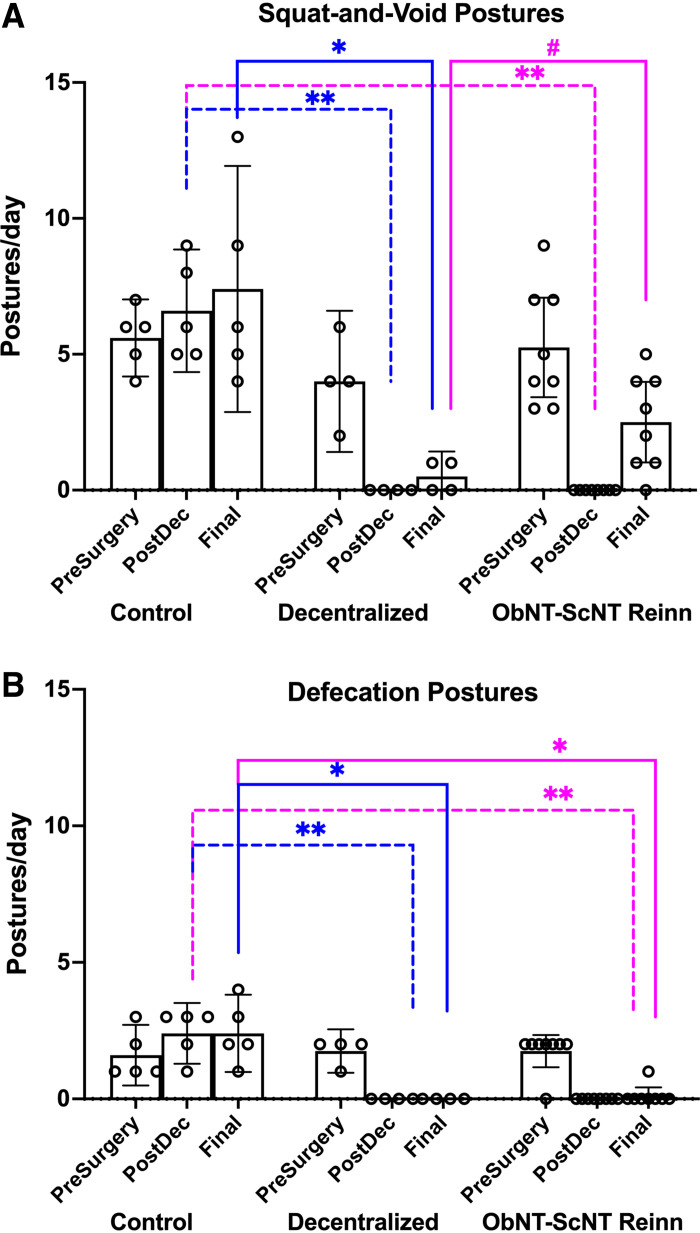
Behavior outcomes. *A*: squat-and-void postures were observed at presurgery (PreSurgery), post decentralization (PostDec), and before euthanasia (Final). *B*: defecation postures at the same time points. A repeated-measures, mixed-effects model was used for both, followed by Tukey–Kramer’s post hoc tests. Means and 95% CI are shown. Dashed lines, group comparisons at PostDec; Solid lines, group comparisons at Final. Pink color denotes comparisons to ObNT-ScNT Reinn group; blue color denotes comparisons to Decentralized group; * and **P < 0.05 and 0.01, respectively, compared with Control; #*P* < 0.05 compared with Decentralized.

**Figure A1.**
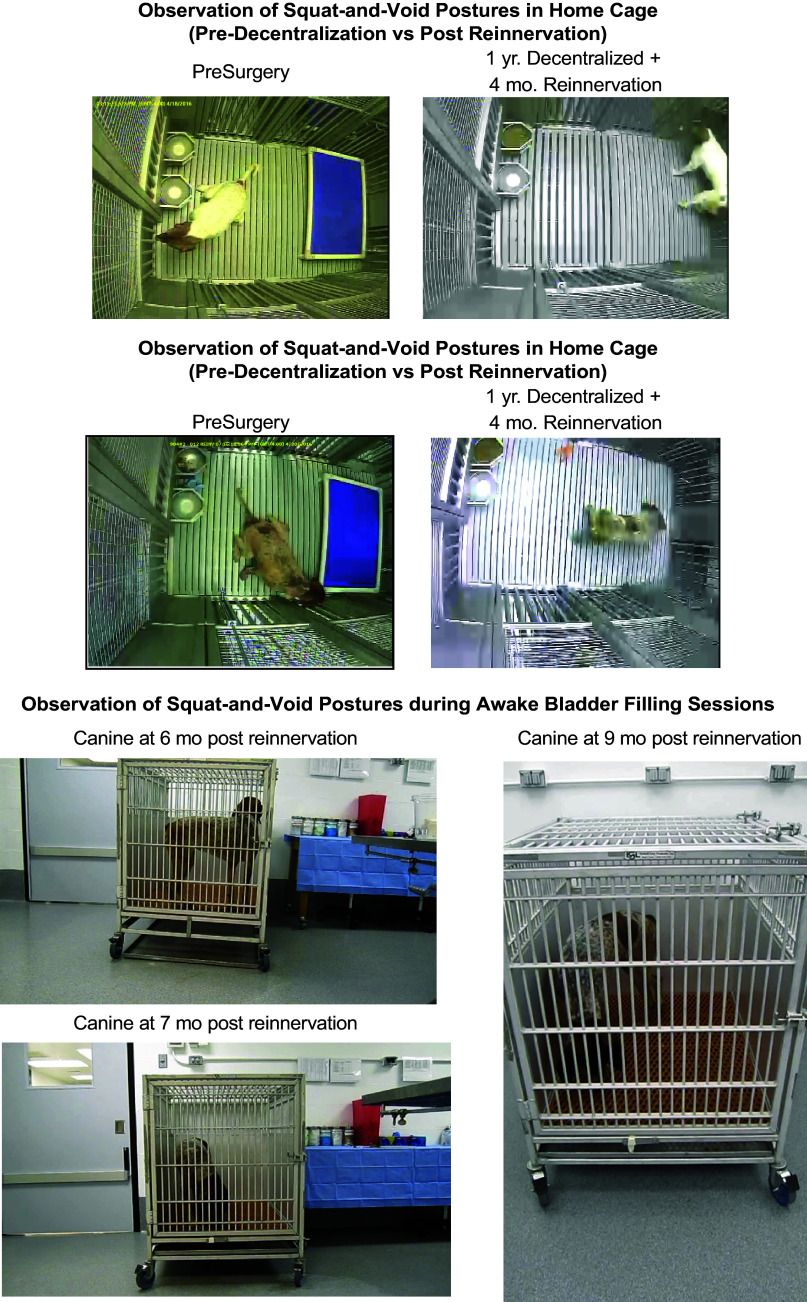
Representative images showing recovery of squat-and-void postures by the Final testing point. An image of a squat-and-void posture in the home cage is shown for a ObNT-ScNT Reinn animal before decentralization (Pre-Surgery). Images of a ObNT-ScNT Reinn animal are then shown at 6, 7, and 9 mo after the reinnervation surgery during awake bladder filling. No squat-and-void posture was seen at 6 mo, a partial squat-and-void posture was seen at 7 mo, while a complete squat-and-void posture was seen at 9 mo after the reinnervation surgery. See also supplemental videos associated with this appendix figure at http://dx.doi.org/10.34944/dspace/9955. These videos show squat-and-void postures in the home cage at 1 yr after decentralization for two animals and at 6, 7, and 9 mo after the reinnervation surgery during awake bladder filling sessions (i.e., awake urodynamics).

Both squat-and-void and defecation postures were reduced post decentralization (Post-Dec) in Decentralized and ObNT-ScNT Reinn animals compared with Control animals (Fig. 2, *A* and *B*). These postures remained reduced in Decentralized animals at final testing compared with Controls (Fig. 2, *A* and *B*), confirming successful long-term decentralization. In contrast, seven of eight ObNT-ScNT Reinn animals showed recovery of squat-and-void postures at the Final testing point, compared with Decentralized animals (Fig. 2*A*; Fig. A1). One ObNT-ScNT Reinn animal even showed voluntary voiding after reinnervation twice following awake bladder filling [see Fig. 5 in our prior pilot study (22); Fig. A1]. Only one of eight ObNT-ScNT Reinn animals showed improvement in defecation postures at final testing (Fig. 2*B*). The mixed-effects model findings for this data, and all subsequent data, are listed in Table A1.

### Stimulation of Upper-to-Midlumbar Spinal Segments Evoked Detrusor Contractility in Reinnervated Animals as Opposed to Sacral Segments in Controls

**Figure 3.**
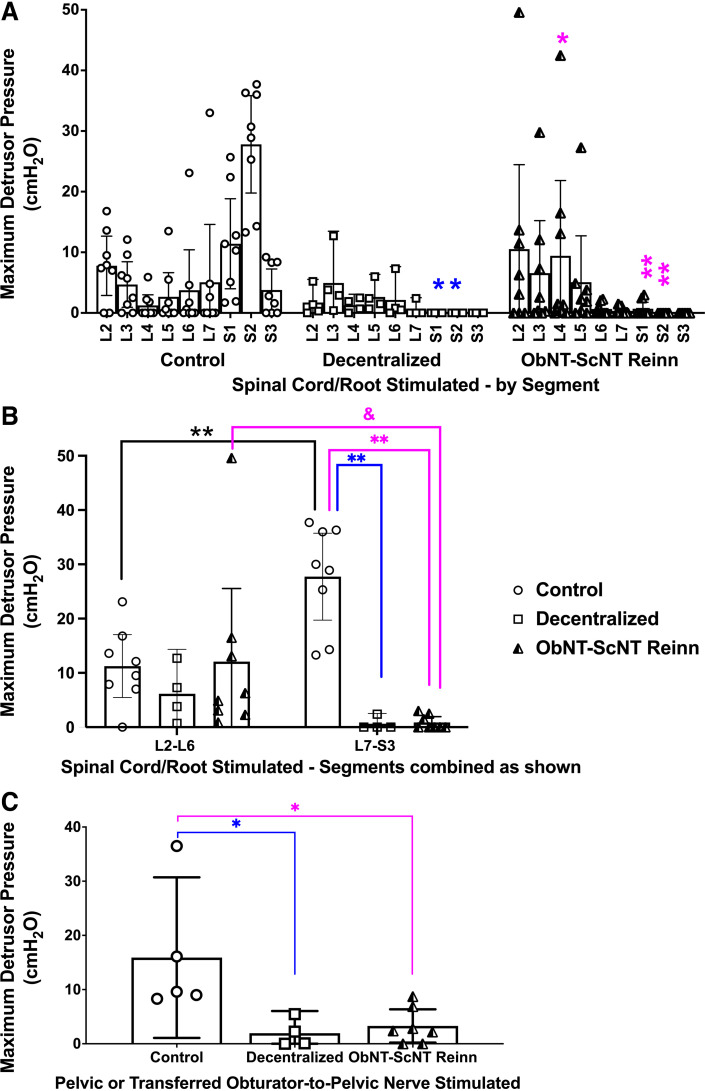
Maximum detrusor pressure (MDP, cmH_2_O) responses during spinal root/cord segmental stimulation or peripheral nerve stimulation (pelvic or ObNT). *A*: MDP responses after spinal root/cord stimulation of individual lumbar (L) to sacral (S) segments from L2-S3. *B*: MDP responses after spinal root/cord stimulation, with segmental responses combined into L2-6 vs. L7-S3, and reanalyzed. *C*: MDP responses after stimulation of pelvic nerve’s vesical branches in Control and Decentralized animals or obturator nerve proximal to the surgical coaptation site in ObNT-ScNT Reinn animals. Repeated-measures mixed-effects models, followed by post hoc tests, were used in *A* and *B*. A Kruskal–Wallis test followed by Dunn’s post hoc tests was used in *C*. Black color in B denotes comparisons between segmental divisions in the Control group; pink color denotes comparisons to ObNT-ScNT Reinn group or comparisons between segmental divisions in the ObNT-ScNT Reinn group; blue color denotes comparisons to Decentralized group; * and **P < 0.05 and 0.01, respectively, compared with Control; &*P* < 0.05 compared with ObNT-ScNT Reinn. Means and 95% CI are shown.

To examine the effects of transferring a lumbar-originating nerve (obturator) to a primarily sacral-originating nerve (pelvic nerve) on bladder contractions, we first examined responses after systematic stimulation of individual root/cord segments from L2 to S3 (Fig. 3*A*). Detrusor pressure responses in S1 and S2 spinal root/cord segments were reduced in Decentralized and ObNT-ScNT Reinn animals, compared with the same segments in Controls (Fig. 3*A*). In contrast, several ObNT-ScNT animals showed a significant increase in L4 evoked detrusor contractility, compared with L4 in Controls (Fig. 3*A*), suggestive of reinnervation of the bladder from the upper to midlumbar cord via the transferred obturator nerve branch.

In Part 2 of this series, we reported MDP responses after combining data into L1-6 and L7-S3 segmental groupings (25). Here, that data was reanalyzed to examine a L2-6 combination to match the origin of obturator nerves more closely in dogs. MDPs were higher in Controls after stimulation of L7-S3 segments than their L2-6 segments (Fig. 3*B*). In contrast, MDPs were lower in Decentralized and ObNT-ScNT Reinn groups after stimulation of L7-S3 segments compared with Control L7-S3 segments (Fig. 3*B*), indicating successful sacral decentralization. In support of the reinnervation strategy, MDPs were higher in ObNT-ScNT Reinn animals after stimulation of L2-6 segments than their L7-S3 segments (Fig. 3*B*).

### ObNT-ScNT Partially Recovered Peripheral Nerve Evoked Detrusor Contractility

In vivo, bladder function after stimulation of pelvic nerves or transferred obturator nerves (stimulated proximal to surgical coaptation site) was reported in Part 2 for all animals used here (25). We present that data again for comparison’s sake (Fig. 3*C*). Briefly, Decentralized animals showed decreased maximum detrusor pressure (MDP) after pelvic nerve stimulation and ObNT-ScNT Reinn animals had decreased MDP after stimulation of the transferred obturator nerves proximal to the surgical coaptation site, compared with Controls (Fig. 3*C*).

### Stimulation of Lower Lumbar-to-Sacral Spinal Segments Evoked Urethra Contractility in Control Animals Only

**Figure 4.**
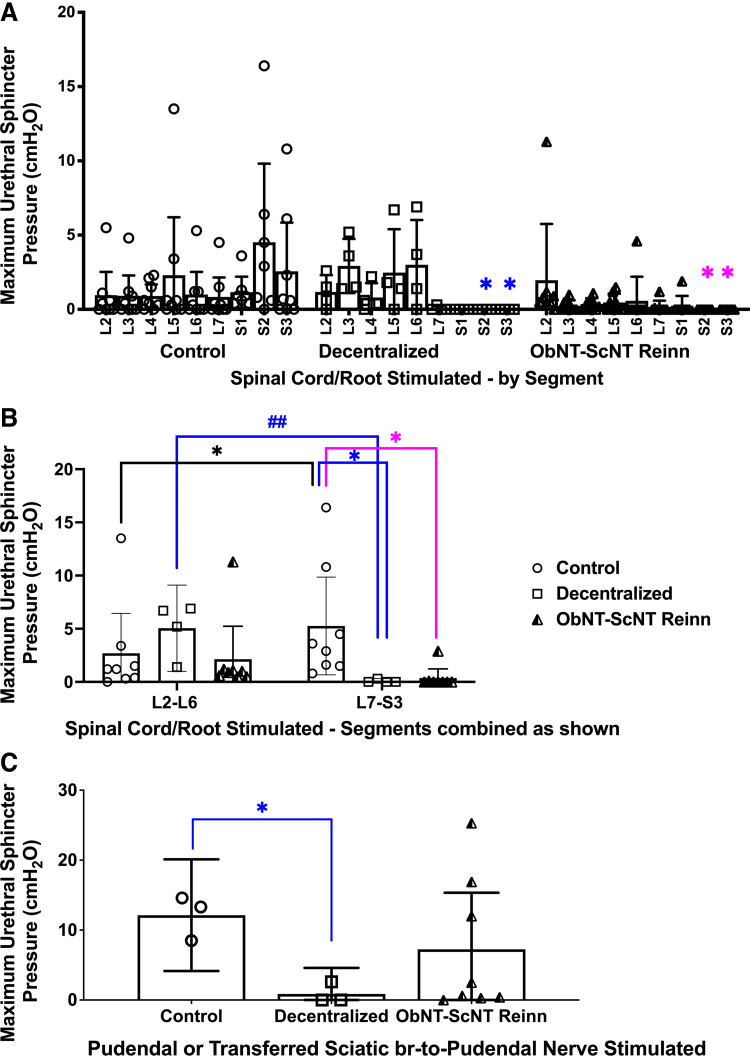
Maximum urethral sphincter pressure (MUSP, cmH_2_O) responses during spinal root/cord segmental stimulation or peripheral nerve stimulation (pudendal or ScNT). *A*: MUSP responses after spinal root/cord stimulation in individual L2-S3 spinal segments. *B*: MUSP responses after spinal root/cord stimulation, with segmental responses combined into L2-L6 vs. L7-S3, and reanalyzed. *C*: MUSP responses after stimulation of the urethral branch of the pudendal nerve in Control and Decentralized animals or after stimulation of the sciatic nerve branch proximal to the surgical coaptation site in ObNT-ScNT Reinn animals. Same statistical methods as defined for Fig. 3. Colors as defined in Figs. 2 and 3 legends; *P < 0.05 compared with Control; ##*P* < 0.01, respectively, compared with Decentralized. Means and 95% CI are shown.

**Figure A2.**
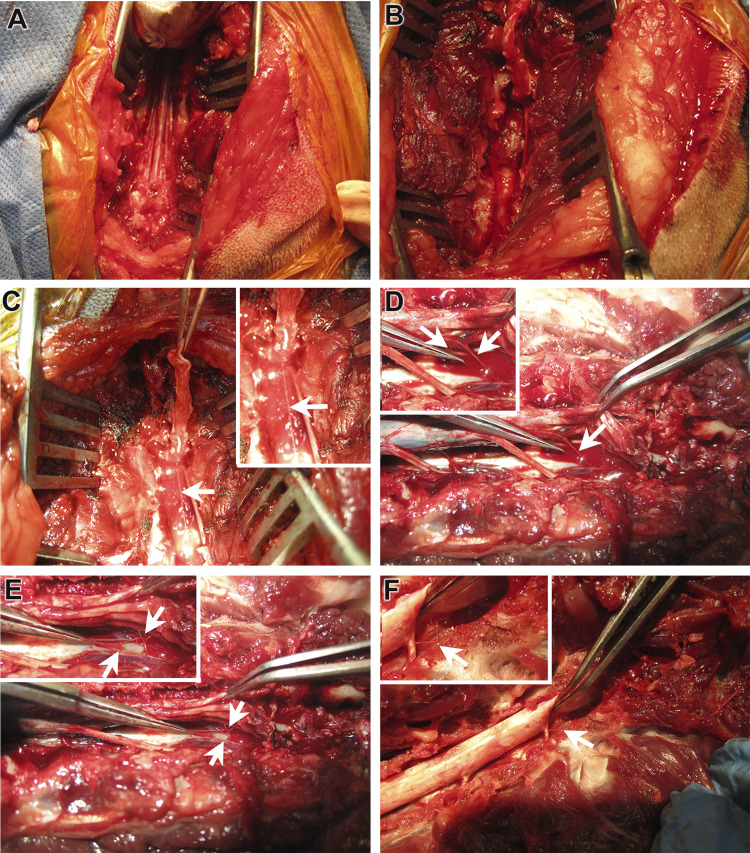
Sprouting of nerves in lumbar ventral cord region. Intact cauda equina roots in lower sacral region, shown after sacral laminectomy (*A*). Sacral roots of cauda equina have been removed for sacral decentralization of urinary end organs (*B*). Sprouts (indicated by white arrow) from lower lumbar region shown below sacral cord (the latter elevated by forceps) in a ObNT-ScNT Reinn animal (*C*). *D*–*F*: Sprouts (indicated by white arrows) from mid or lower lumbar region shown below sacral cord (the latter elevated by forceps) in two different Decentralized animals, with *D* and *E* showing one Decentralized animal and *F* showing a second. *Insets*: close-up. Spinal roots (ventral and dorsal combined as they are still in their dural sac) are indicated by black arrows.

When recording bladder stimulation findings, we simultaneously recorded maximum urethral sphincter pressure (MUSP) responses when stimulating individual root/cord segments (Fig. 4*A*). MUSP was reduced in Decentralized and ObNT-ScNT Reinn animals when S2 and S3 segments were stimulated compared with these segments in Controls (Fig. 4*A*). MUSP responses were then combined into L2-6 versus L7-S3 segmental combinations and reanalyzed. MUSP was higher in L7-S3 segments of Controls compared with their L2-L6 segments (Fig. 4*B*) and compared with responses of L7-S3 segments in Decentralized and ObNT-ScNT Reinn animals (Fig. 4*B*), supportive of successful sacral denervation of the urethra in the latter two groups. Interestingly, in Decentralized animals, MUSP responses were higher in L2-6 segments than in their L7-S3 segments (Fig. 4*B*), suggestive of sprouting from these segments in Decentralized animals. Gross examination of the spinal cord during tissue collection revealed small neurite sprouts exiting from the ventral region of lumbar and sacral cord regions of several of the Decentralized animals (Fig. A2, *D*–*F*). Similar sprouting was also observed in some of the ObNT-ScNT Reinn animals (Fig. A2*C*). These small neurites took a variety of routes caudally in the vertebral column.

### Reinnervated Animals Showed Some Improvement in Peripheral Nerve Evoked Urethral Sphincter Contractility

We have reported MUSP responses after stimulation of transferred sciatic-to-pudendal nerve for three ObNT-ScNT Reinn animals in our prior pilot study (22). Those findings are expanded here to show results from 8 total ObNT-ScNT Reinn animals (Fig. 4*C*). MUSP responses with pudendal nerve stimulation were reduced in Decentralized animals (Fig. 4*C*) compared with Controls. In contrast, MUSP responses in ObNT-ScNT Reinn animals after stimulation of their transferred sciatic-to-pudendal nerves were similar to Controls (Fig. 4*C*), supportive of reinnervation, although data in this group was driven mainly by data from three ObNT-ScNT Reinn animals.

### Anal Sphincter Evoked Contractility Was Reduced in Decentralized and Reinnervated Groups

**Figure 5.**
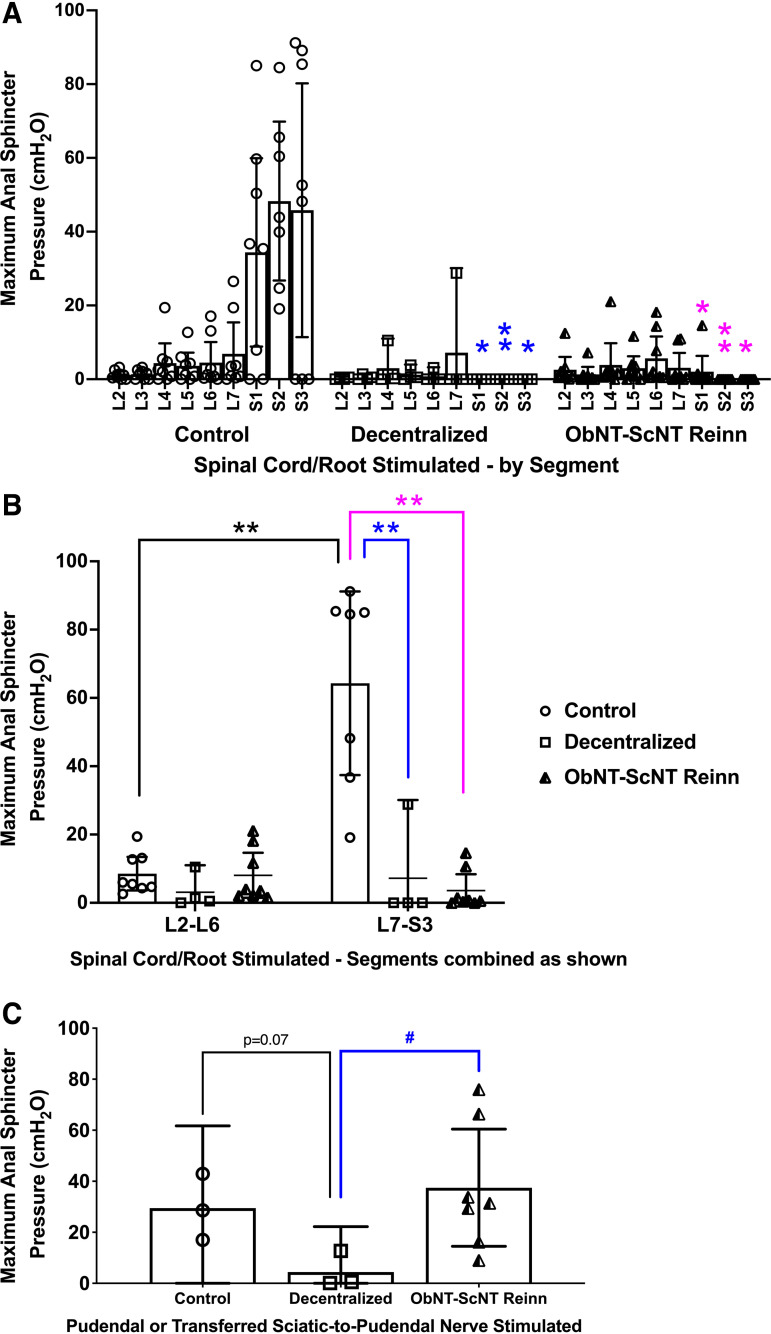
Maximum anal sphincter pressure (MASP) responses during spinal root/cord segmental stimulation or peripheral nerve stimulation (pudendal or ScNT). *A*: MASP responses after spinal root/cord segmental stimulation in individual L2-S3 segments. *B*: MASP responses after spinal root/cord stimulation, with the segmental responses combined into L2-L6 vs. L7-S3, and reanalyzed. *C*: MASP responses after stimulation of the anal branch of the pudendal nerve in Control and Decentralized animals or after stimulation of the sciatic nerve branch proximal to the surgical coaptation site in ObNT-ScNT Reinn animals. Same statistical methods as defined for Fig. 3. Colors as defined in Figs. 2 and 3 legends; * and **P < 0.05 and 0.01, respectively, compared with Control; #*P* < 0.05 compared with Decentralized. Means and 95% CI are shown.

We also simultaneously recorded maximal anal sphincter pressure (MASP) responses when stimulating individual root/cord segments (Fig. 5*A*). MASP was dramatically reduced in Decentralized and ObNT-ScNT Reinn animals when S1-3 root/cord segments were stimulated compared with Controls (Fig. 5*A*), in support of decentralization of the anal sphincter by the decentralization procedure. When this MASP data was combined into L2-6 and L7-S3 combinations and reanalyzed, MASP was highest in Control animals after stimulation of L7-S3 compared with their L2-6 results and compared with the L7-S3 results of Decentralized and ObNT-ScNT Reinn animals (Fig. 5*B*).

### Reinnervation Leads to Improved Peripheral Nerve-Evoked Anal Sphincter Contractility

We have reported MASP responses after stimulation of transferred sciatic-to-pudendal nerve for three ObNT-ScNT Reinn animals in our prior pilot study (22). Those findings are expanded here to show results from eight total ObNT-ScNT Reinn animals (Fig. 5*C*). MASP responses after stimulation of the transferred sciatic nerve were higher in ObNT-ScNT Reinn animals compared with stimulation of the decentralized pudendal nerve in Decentralized animals (Fig. 5*C*). All eight ObNT-ScNT Reinn animals showed elevated MASP responses similar to Controls, suggestive of regrowth across the nerve coaptation site (Fig. 5*C*).

### Retrograde Labeling in Ventral Horns Supports Growth of Motor Axons to Bladder and Urethra from Upper Lumbar Segments following Reinnervation

**Figure 6.**
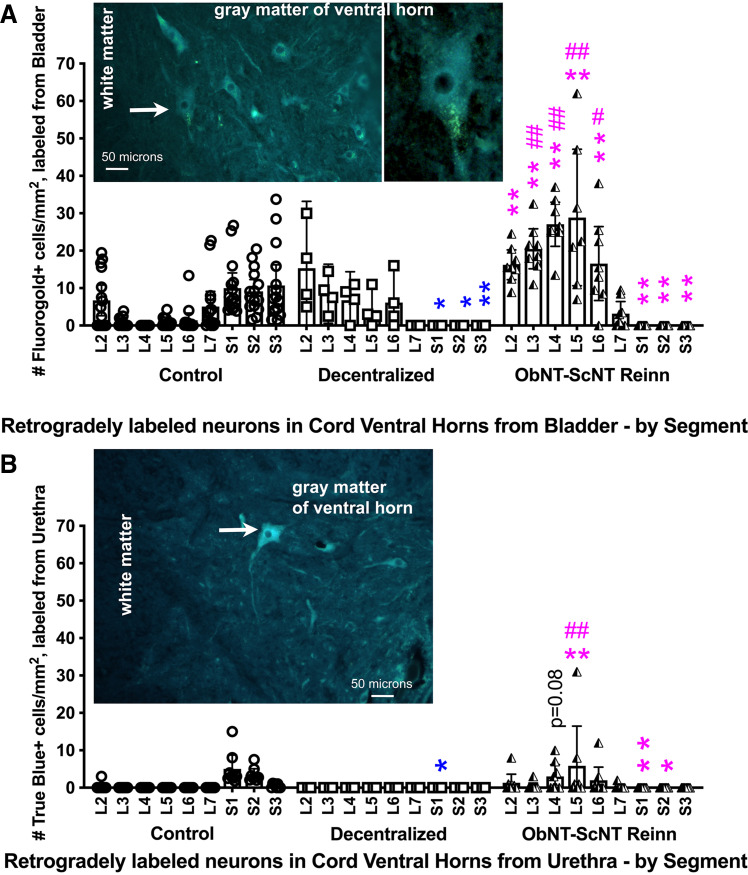
Retrograde dye-labeled neurons in ventral horns of lumbar (L) to sacral (S) spinal cord segments after injections of dye into bladder or urethra. *A*: after injections of dye into bladder, number of Fluoro-Gold-labeled neurons per mm^2^ in spinal cord ventral horns. Examples of Fluoro-Gold-labeled neurons are shown in the *insets* (visible as neurons with punctate gold to white dots in their cytoplasm); the arrow indicates the neurons enlarged in additional *inset*. *B*: after injections of dye into urethra, number of True Blue-labeled neurons per mm^2^ in spinal cord ventral horns are shown. An example of a True Blue-labeled neurons is shown in the *inset* (arrow; visible as blue-labeled neuron). A repeated-measures, mixed-effects model was used for each set of analyses. Simple differences were also examined using least squares means with adjustments for multiple comparisons using Tukey–Kramer method. Colors as defined in Fig. 2 and 3 legends; * and **P < 0.05 and 0.01, respectively, compared with Control; # and ##*P* < 0.05 and 0.01, respectively, compared with Decentralized. Means and 95% CI are shown.

Retrograde tracing methods were used to further examine the efficacy of both decentralization and reinnervation surgeries. Fluoro-gold was injected into the bladder at four sites around each ureteral orifice and allowed to retrogradely travel to the spinal cord during a 3-wk recovery period. After spinal cord collection and fixation, an assessment of numbers of Fluoro-gold dye-labeled neurons in sectioned spinal cord segments showed lowered numbers of Fluoro-gold-labeled neurons in S1-S3 ventral horns in Decentralized and ObNT-ScNT Reinn animals compared with Controls (Fig. 6*A*), indicative of successful sacral decentralization of the bladder in the Decentralized and ObNT-ScNT Reinn animals. In contrast, numbers of Fluoro-gold-labeled neurons in L2-6 ventral horn segments were higher in ObNT-ScNT Reinn animals compared with Controls (Fig. 6*A*). They were also higher in L3-6 ventral horn segments in ObNT-ScNT Reinn animals compared with Decentralized animals (Fig. 6*A*). Representative images for Fluoro-Gold-labeled neurons in spinal cord ventral horns are shown in *insets* in Fig. 6*A.* These latter results are supportive of reinnervation of the bladder across the obturator to pelvic nerve coaptation site.

At 3 wk before euthanasia, True Blue was injected into the urethra. An assessment of numbers of labeled neurons from the urethra in spinal cord ventral horns showed that labeled neurons were reduced in S1 ventral horn segment of Decentralized animals, and in S1 and S2 ventral horn segments of ObNT-ScNT Reinn animals compared with Controls (Fig. 6*B*), indicative of successful sacral decentralization of the urethra. In contrast, there were more labeled neurons in the L5 segments of ObNT-ScNT Reinn animals compared with the other groups and a trend toward an increase in the L4 segment of ObNT-ScNT Reinn animals compared with Controls (*P* = 0.08), the former supportive of reinnervation of the urethra across the sciatic branch to pudendal nerve coaptation site. Representative images for True Blue-labeled neurons in spinal cord ventral horns are shown in *insets* in Fig. 6*B.*

### Reinnervated Animals Had More Retrograde Labeled Neurons from the Bladder in Laminae VII, VIII, and IX of Upper Lumbar Segments than the Other Groups

**Figure 7.**
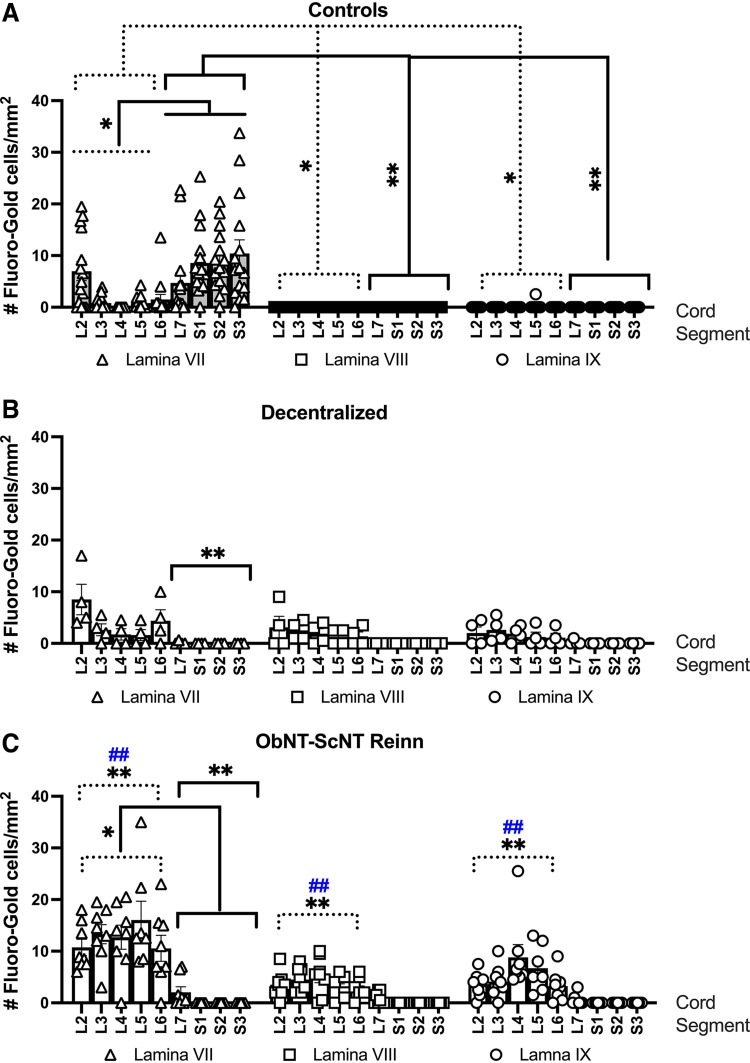
After injections of dye into the bladder, numbers of Fluoro-Gold-labeled neurons per mm^2^ in spinal cord ventral horns divided into laminar (VII, VIII, and IX) and segmental (L2-S3) location are shown. *A*: Control, *B*: Decentralized, and *C*: ObNT-ScNT Reinn animals. Analyses of retrogradely labeled neurons in the ventral horns were performed for each dye using a repeated-measures mixed-effects model and three factors: surgical group, combined segments of L2-L6 vs. L7-S3, and lamina. This was followed by Tukey–Kramer multiple comparison post hoc tests. Dotted lines delineating segments L2-L6; solid black lines delineating segments L7-S3; Colors as defined in Fig. 2 and 3 legends; * and **P < 0.05 and 0.01, respectively, compared with Control; ##*P* < 0.01 compared with Decentralized. Means and 95% CI are shown.

We next assessed whether the laminar and segmental location of retrogradely labeled neurons innervating the bladder changed with the reinnervation strategy (Fig. 7, *A*–*C*). For simplification, spinal segments were combined into L2-6 and L7-S3 groupings for the statistical analyses. Control animals had more labeled neurons from the bladder (i.e., Fluoro-Gold-labeled neurons) in lamina VII of L7-S3 ventral horns versus lamina VII of L2-L6 (Fig. 7*A**, left*) and compared with laminae VIII and IX of L7-S3 segments (Fig. 7*B*, *middle* and *right*). Control animals also had more labeled neurons in lamina VII in L2-L6 ventral horns compared with laminae VIII and IX in these L2-L6 segments (Fig. 7*A*). Decentralized animals showed no within-group lamina or segmental differences (Fig. 7*B*). In contrast, ObNT-ScNT Reinn animals had more labeled neurons in lamina VII of L2-L6 ventral horns versus lamina VII in L7-S3 (Fig. 7*C*, *left*), supportive of reinnervation of the bladder through the transferred Obturator to pelvic nerve. ObNT-ScNT Reinn animals also showed more labeled neurons in lamina VII of L2-6 ventral horns versus in laminae VIII and IX of L2-L6 segments (*P* < 0.05 each, not depicted in Fig. 7*C* for simplification of the figure).

When comparing across surgical groups, Decentralized animals had fewer retrogradely labeled cells in lamina VII of L7-S3 ventral horns versus Controls (Fig. 7*B*) as did ObNT-ScNT Reinn animals (Fig. 7*C*). In contrast, ObNT-ScNT Reinn animals had more retrogradely neurons in lamina VII of L2-L6 ventral horn segments than the same lamina and segments of Control and Decentralized animals and more labeled cells in laminae VIII and IX of L2-L6 ventral horn segments than the same lamina and segments of Control and Decentralized animals (Fig. 7*C* vs. Fig. 7, *A* and *B*).

The three-way mixed-effects model findings are provided in Table A1.

### There Was Low Regrowth from Spinal Cord to the Urethra in Reinnervated Animals

**Figure 8.**
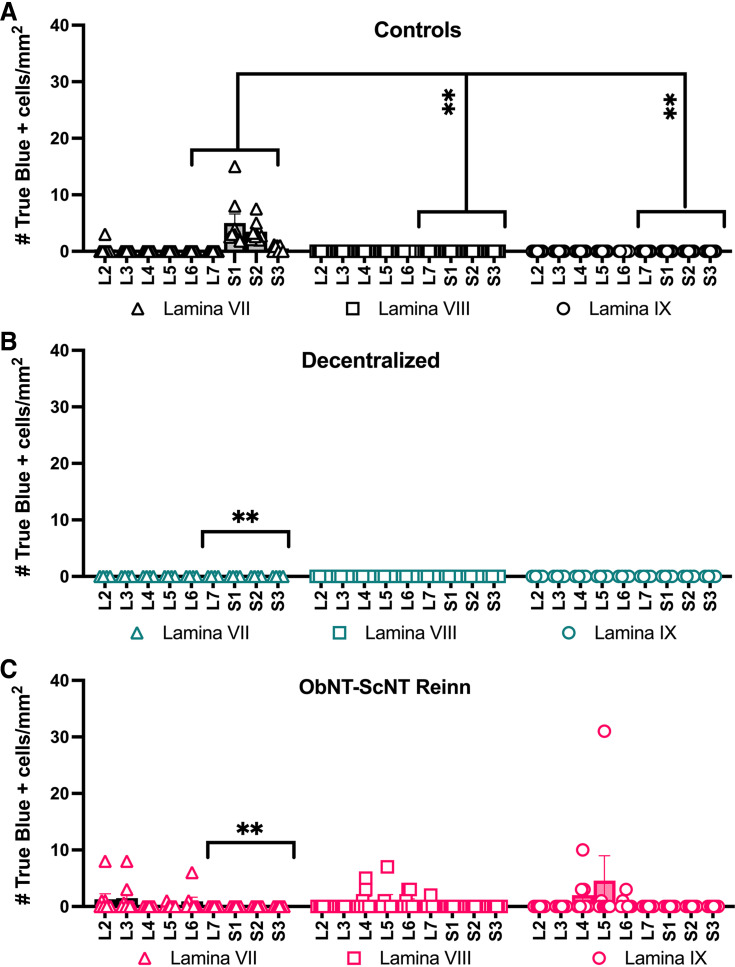
The number of True Blue-labeled neurons per mm^2^, after injections of dye into the urethra, in spinal cord ventral horns presented by segment (L2-S3) and lamina (VII, VIII, and IX). *A*: Control animals, *B*: Decentralized animals, and *C*: ObNT-ScNT Reinn animals. Same statistical methods as defined for Fig. 7. Solid black lines delineating segments L7-S3; Colors as defined in Figs. 2 and 3 legends; **P < 0.01, respectively, compared with Controls. Means and 95% CI are shown.

The lamina location of retrogradely labeled neurons from the urethra (i.e., True Blue-labeled neurons) was also analyzed after division of data into L2-6 versus L7-S3 segmental groupings (Fig. 8) and then by three-way repeated-measures mixed-effects modeling. Control animals had more labeled neurons from the urethra in lamina VII of L7-S3 segments compared with lamina VIII and IX in the same segments (i.e., L7-S3; Fig. 8*A*). Few to no labeled neurons from the bladder were seen in any lamina or segment in Decentralized animals; therefore, no within-group differences were seen (Fig. 8*B*). Yet, the ObNT-ScNT Reinn animals showed relatively more labeling (although still low) in each laminae (VII, VIII, and IX) in L2-6 ventral horn segments, than in these same laminae in L7-S3 segments (Fig. 8*C*).

When comparing across surgical groups, both Decentralized and ObNT-ScNT Reinn animals showed fewer retrogradely labeled neurons from the urethra in lamina VII in L7-S3 segments compared with lamina VII in L7-S3 segments of Controls (Fig. 8, *B* and *C*). In contrast, ObNT-ScNT Reinn animals showed more labeling in each lamina (VI, VIII, and IX) of L2-6 ventral horn segments compared with that seen in these same lamina and L2-6 segments of Control and Reinnervated groups (Fig. 8*C* vs. Fig. 8, *A* and *B*).

The three-way, mixed-effects model findings are provided in Table A1.

### Correlations between Outcomes

To determine if the outcomes were linked and if the responsiveness of one outcome was linked to responsiveness to another outcome, several correlations were performed.

**Figure A3.**
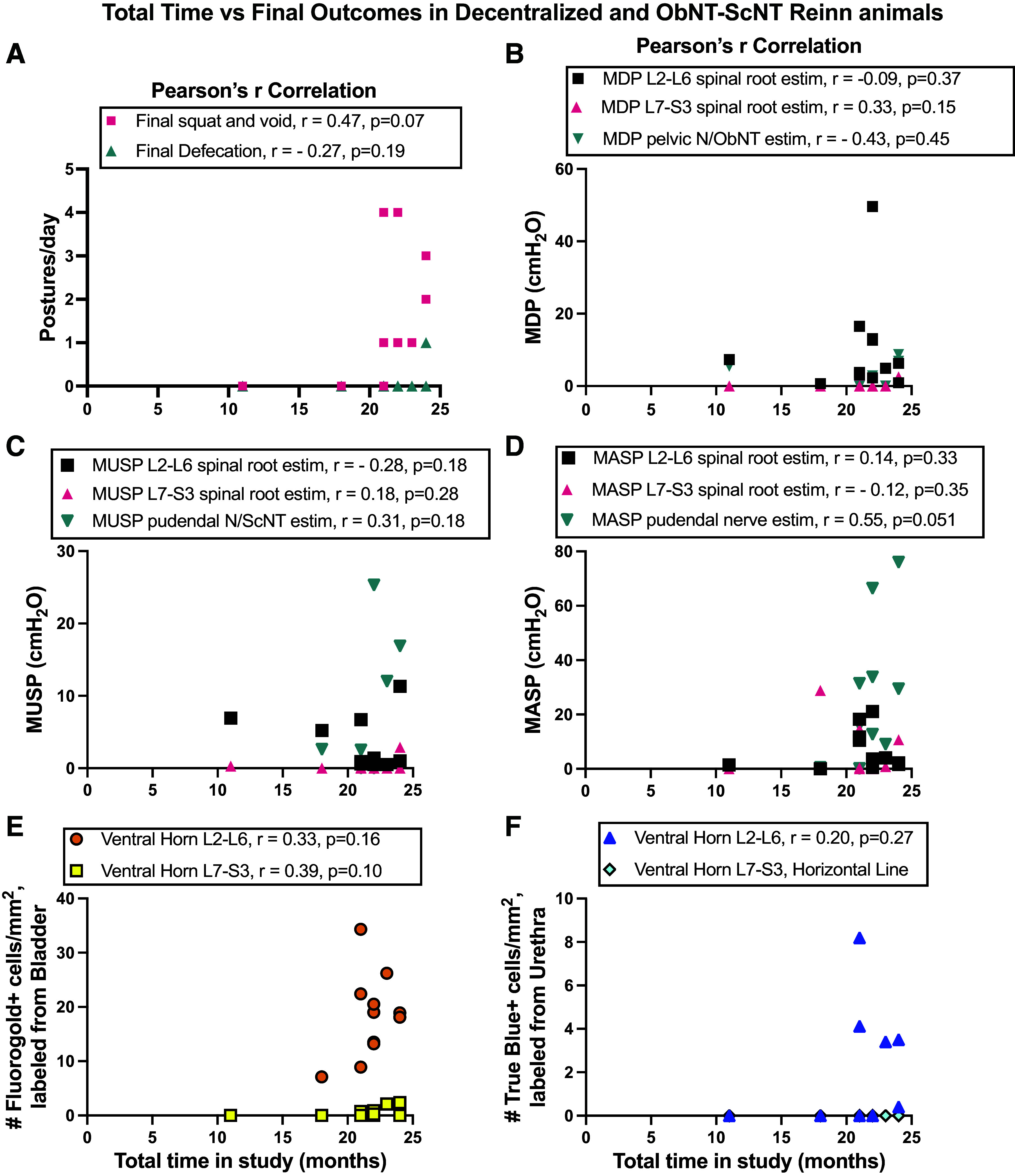
Correlations between total time in study vs. final outcomes in Decentralized and ObNT-ScNT Reinn animals. Correlations between total time in study vs. the following: final squat-and-void postures and final defecation postures/day (*A*); MDP, MUSP or MASP responses after stimulation of nerves or spinal root combinations indicated (*B*–*D*); numbers of Fluoro-Gold (FG)-labeled neurons in ventral horn lumbar segments indicated (*E*); and numbers of True Blue (TB)-labeled neurons in ventral horn lumbar indicated (*F*). No significant correlations were observed.

**Figure A4.**
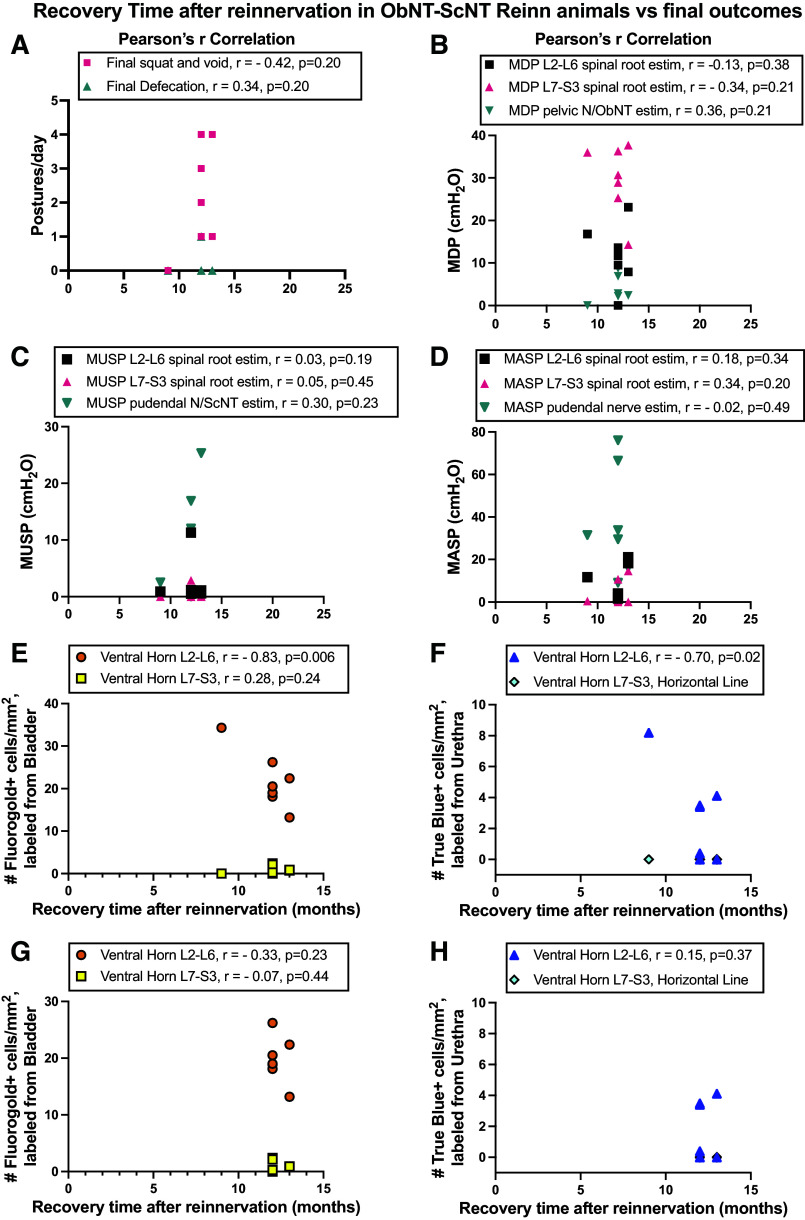
Correlations between recovery time between reinnervation and euthanasia vs. final outcomes in ObNT-ScNT Reinn animals. Correlations between this recovery time vs. the following: final squat-and-void and defecation postures/day (*A*); MDP, MUSP, or MASP responses after stimulation of nerves or spinal root combinations indicated (*B*–*D*); numbers of Fluoro-Gold-labeled neurons in lumbar spinal cord laminae indicated (*E*); and numbers of True Blue (TB)-labeled neurons in ventral horn lumbar segments indicated (*F*). *G* and *H*: same data as shown in *E* and *F* except with the one 9-mo recovery ObNT-ScNT Reinn animal excluded.

**Figure A5.**
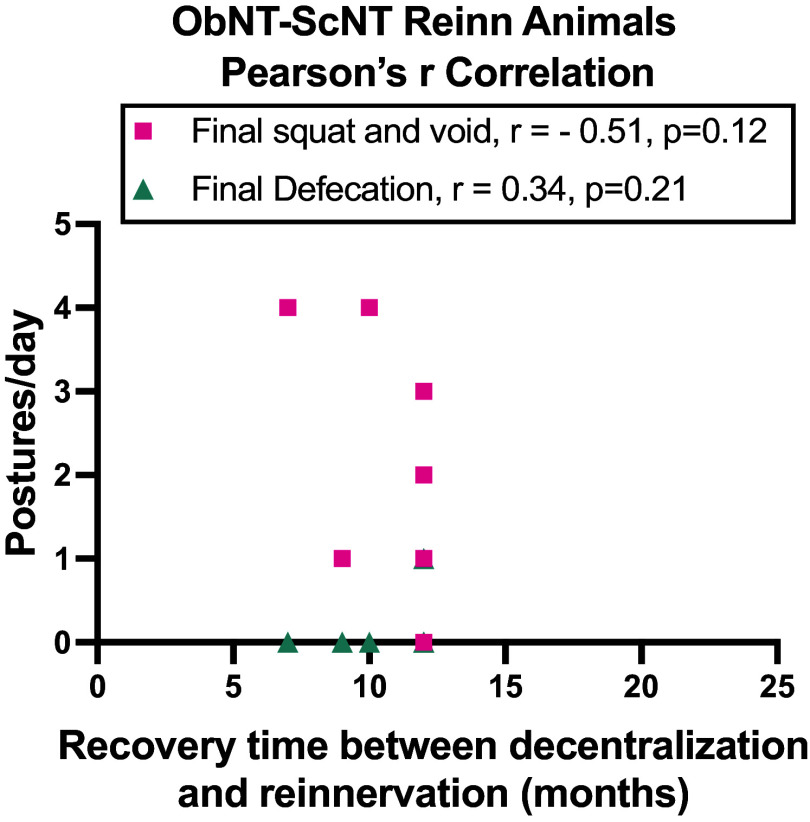
Correlation between recovery time between decentralization and reinnervation surgeries and postures/day in ObNT-ScNT Reinn animals. No significant correlation was observed.

First, we correlated total time in study of Decentralized and ObNT-ScNT Reinn animals with final outcomes (Fig. A3). There were no significant correlations. We also correlated the recovery time after reinnervation in the ObNT-ScNT Reinn animals with their final outcomes (Fig. A4). When the one ObNT-ScNT Reinn animal that recovered 9 mo after reinnervation, a strong negative correlation was seen between recovery time and Fluorogold and True Blue-labeled neurons in the L2-L6 ventral horn segments due to the high number of labeled neurons in this animal (*r* = −0.83 and *r* = −0.70, Fig. A4, *E* and *F*, respectively). No other correlations were observed. However, when data from that animal was excluded, those correlations were lost (*r* = −0.33 and *r* = 0.15, Fig. A4, *G* and *H*, respectively). We next correlated the recovery time of ObNT-ScNT animals between their decentralization and reinnervation surgeries with their final postures/day (Fig. A5). There was no significant correlation.

**Figure 9.**
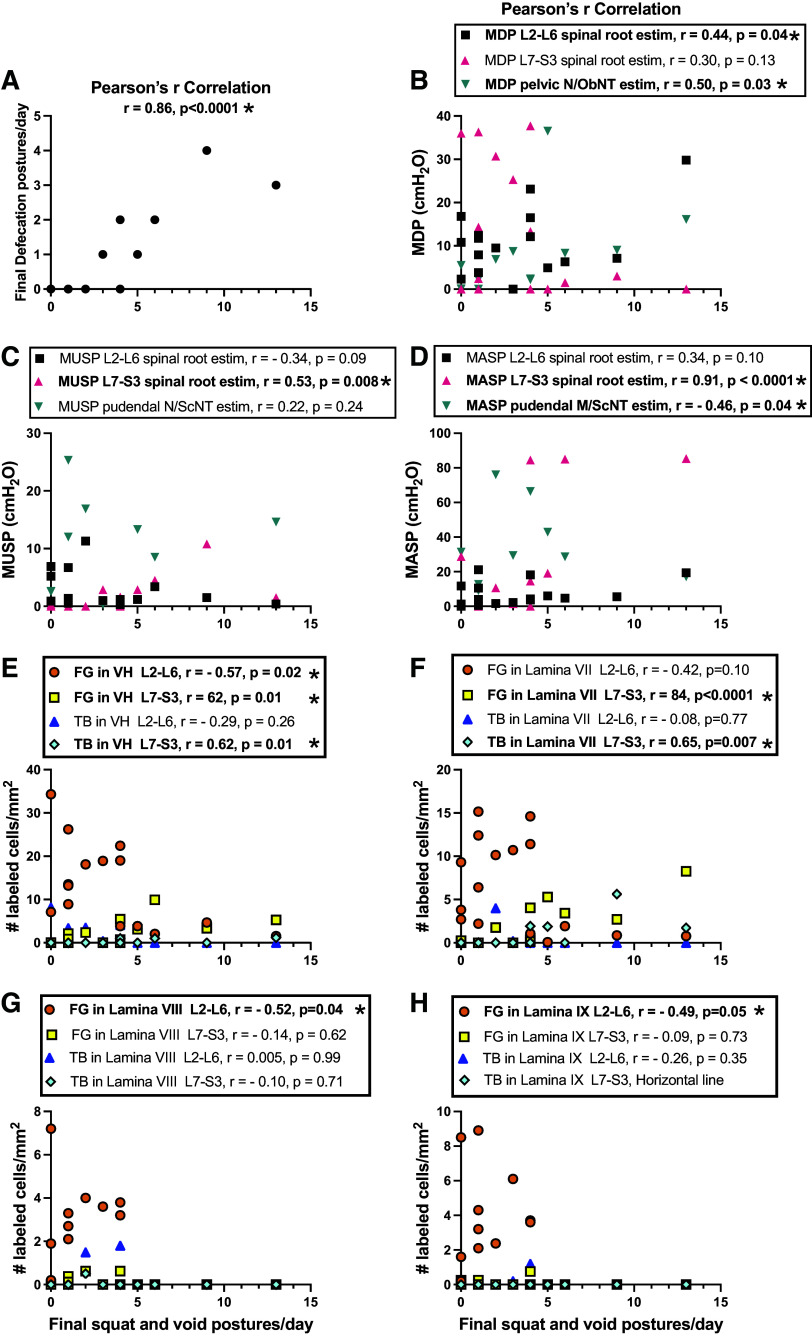
Correlations between final squat-and-void postures/day and other final outcomes from Controls, Decentralized, and ObNT-ScNT Reinn animals. Correlation results between final squat-and-void postures/day and: final defecation postures/day (*A*); MDP after stimulation of roots or nerves indicated (*B*); MUSP after stimulation of roots or nerves indicated (*C*); MASP after stimulation of roots or nerves indicated (*D*); and number of Fluoro-Gold (FG) and True Blue (TB) labeled neurons in L2-L6 segments’ or L7-S3 segments’ ventral horns (VH, with results for lamina VII-IX averaged; *E*), lamina VII (*F*), lamina VIII (*G*), and lamina IX (*H*). Significant findings are bolded and indicated with an asterisk.

Next, we correlated squat-and-void postures at the final testing point with the other final outcomes in all animals (Fig. 9, *A*–*H*). Final squat-and-void postures per day correlated strongly and positively with the final defecation postures per day (Fig. 9*A*). These behaviors also correlated moderately and positively with: *1*) MDP responses after stimulating L2-L6 spinal roots and pelvic nerve or proximal portion of the transferred obturator nerve (Fig. 9*B*); *2*) MUSP responses after stimulating L7-S3 spinal roots (Fig. 9*C*); *3*) number of Fluoro-Gold and True Blue-labeled neurons (from bladder and urethra, respectively) in L7-S3 ventral horns (data from lamina VII-IX averaged; Fig. 9*E*); and *4*) number of True Blue-labeled neurons in lamina VII of L7-S3 segments (Fig. 9*F*). These behaviors also correlated strongly and positively with MASP responses after stimulating L7-S3 spinal roots (Fig. 9*D*) and number of Fluoro-Gold-labeled neurons in lamina VII of L7-S3 segments (Fig. 9*F*). In addition, these behaviors correlated moderately and negatively with MASP responses after stimulating pudendal nerves or proximal portion of the transferred sciatic nerve (Fig. 9*D*) and number of Fluoro-Gold-labeled neurons in L2-L6 ventral horns (laminae VII-IX averaged; Fig. 9*E*), and in laminae VIII and IX of L2-L6 segments (Fig. 9*G* and *H*). In summary, positive associations were found between squat-and-void postures and spinal root and peripheral nerve-evoked responses after obturator nerve transfer (Fig. 9*B*) and in Fluoro-Gold-labeled neurons in L2-S3 segments (Fig. 9, *E*, *G*, and *H*), each in support of the formation of a new neuronal pathway from the obturator nerve transfer. Correlations between squat-and-void postures and MUSP and MASP evoked responses after stimulating L7-S3 spinal roots (Fig. 9, *C* and *D*), and between these postures and labeled neurons in L7-S3 segments (Fig. 9, *E* and *F*), were observed as expected since the bladder and urethra receive innervation from these segments in Control animals.

**Figure A6.**
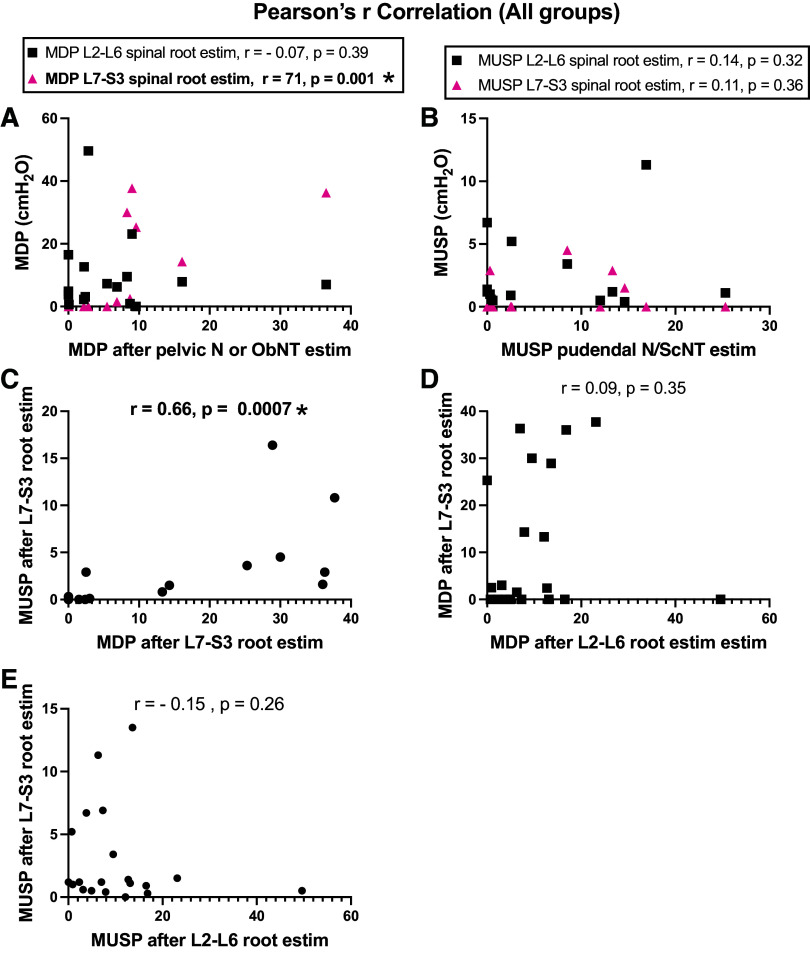
Correlations between the electrophysiological results from all groups (Controls, Decentralized, and ObNT-ScNT Reinn animals). Correlations between MDP after stimulation of nerves or roots indicated (*A*). Correlations between MUSP after stimulation of nerves or roots indicated (*B*). Correlation of MDP vs. MUSP after stimulation of L7-S3 spinal roots (*C*). Correlation of MDP after stimulation of L2-L6 vs. L7-S3 spinal roots (*D*). Correlation of MUSP after stimulation of L2-L6 vs. L7-S3 spinal roots (*E*). VH, ventral horn; pelvic N/ObNT, pelvic nerve and proximal portion of transferred obturator nerve branch; pudendal N/ScNT, pudendal nerve and proximal portion of transferred sciatic nerve branch. Significant findings are bolded and indicated with an asterisk.

Then, we correlated the various electrical stimulation responses with each other (Fig. A6). MDP responses after stimulation of the pelvic nerve or proximal portion of the transferred obturator nerve correlated moderately and positively with MDP responses after L7-S3 spinal root stimulation (*r* = 0.71, *P* = 0.001), although not with responses after L2-L6 spinal root stimulation (*r* = −0.07, *P* = 0.39; Fig. A6*A*). MUSP responses after stimulation of the pudendal nerve or proximal portion of the transferred sciatic nerve did not correlate with L2-L6 or L7-S3 spinal root stimulation responses (Fig. A6*B*). Yet, MDP versus MUSP responses after L7-S3 spinal root stimulation correlated moderately and positively (*r* = 0.66, *P* = 0.0007; Fig. A6*C*). There were no significant correlations between MDP or MUSP responses after L2-L6 spinal root stimulation versus L7-S3 spinal root stimulation (Fig. A6, *D* and *E*).

**Figure 10.**
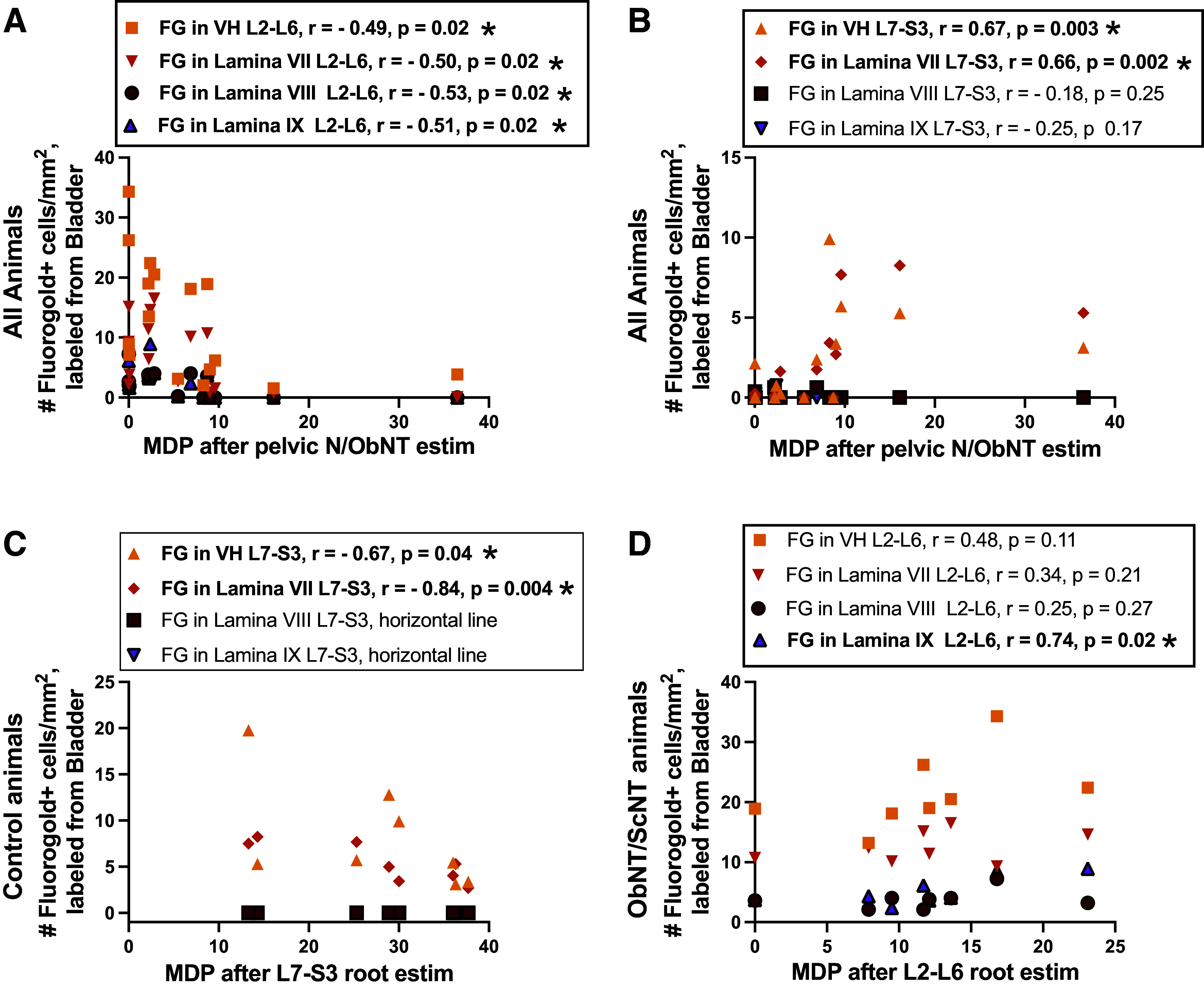
Correlations between maximum detrusor pressure (MDP)-evoked results and number of Fluoro-Gold-labeled neurons in spinal cord. *A*: correlations between Fluoro-Gold-labeled neurons in all groups in L2-L6 segments’ ventral horns (VH, with results for laminae VII-IX averaged) and laminae VII-IX, individually, versus MDP after stimulation of pelvic N/ObNT. *B*: correlations between Fluoro-Gold-labeled neurons in all groups L7-S3 segments’ ventral horns (VH, results for laminae VII-IX averaged) and laminae VII-IX, individually, vs. MDP after stimulation of pelvic N/ObNT. *C*: correlations between Fluoro-Gold-labeled neurons in Control animals in ventral horns (VH, results for laminae VII-IX averaged) and laminae VII-IX, individually, vs. their MDP after L7-S3 spinal root stimulation. *D*: correlations between Fluoro-Gold-labeled neurons in ObNT/ScNT Reinn animals in ventral horns (VH, results for laminae VII-IX averaged) and laminae VII-IX, individually, vs. their MDP after L2-L6 spinal root stimulation. Significant findings are bolded and indicated with an asterisk.

**Figure A7.**
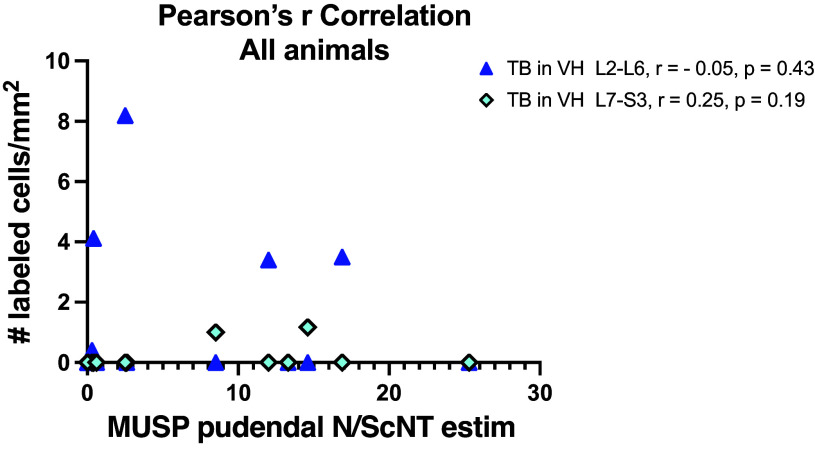
Correlations between maximum urethral sphincter pressure (MUSP) responses after stimulation of the pudendal N/ScNT (pudendal nerve and proximal portion of transferred sciatic nerve branch) vs. number of True Blue (TB)-labeled neurons in ventral horns (VH) of L2-L6 or L7-S3 segments. Results for all animals per group provided. No significant correlations were observed.

Finally, we correlated the electrical stimulation responses with the location of Fluoro-Gold-labeled neurons in the spinal cord (segmental and laminar location) after injections into the bladder (Fig. 10) and MUSP responses and the location of True blue-labeled neurons in the spinal cord (Fig. A7). In all animals, MDP responses after stimulation of the pelvic nerve or proximal transferred obturator nerve correlated with Fluoro-Gold dye results in L2-L6 segments, specifically with ventral horns in general (with laminar data combined), laminae VII, VIII, and IX (Fig. 10*A*). Also in all animals in the study, these peripheral nerves evoked MDP responses correlated with Fluoro-Gold results in L7-S3 ventral horns in general and lamina VII (Fig. 10*B*). We next examined for correlations between spinal root evoked MDP responses and the location of labeled neurons in Controls separately from ObNT-ScNT Reinn animals (Fig. 10, *C* and *D*). In Controls, there were significant correlations with Fluoro-Gold-labeled neurons in L7-S3 ventral horns in general and lamina VII (Fig. 10*C*), the latter matching the origin of the pelvic nerve. In ObNT-ScNT Reinn animals, there were significant correlations with the location of labeled neurons in L2-L6 lamina IX (Fig. 10*D*). There were no significant correlations between MUSP responses and the location of True blue-labeled neurons in the spinal cord (Fig. A7). In summary, correlations were found between MDP evoked responses and Fluoro-Gold-labeled cells in L2-L6 ventral horns, lamina VIII and IX (Fig. 10, *A* and *D*), in support a return of bladder function as a consequence of the obturator nerve transfer (which originates from upper to mid lumbar segments), and innervation of the bladder by somatic motor axons that originally innervated appendicular muscles (axons that originate in lamina IX).

## DISCUSSION

We sought to examine the effectiveness of bilateral somatic nerve transfer to the vesical branch of the pelvic nerve and pudendal nerve after a year-long decentralization by examining micturition and defecation behaviors, functional electrical stimulation of spinal roots/segments of L2-S3 and transferred nerves, and retrograde labeling from the bladder and urethra to ventral horns of the spinal cord. Seven of 8 (88%) of the ObNT-ScNT Reinn animals showed a return of squat-and-void postures by 9–10 mo after reinnervation surgery. One even showed voluntarily voiding twice following awake bladder filling [see Fig. A1, and Fig. 5 in our prior pilot study (22)]. Spinal root/cord stimulation in ObNT-ScNT Reinn animals evoked bladder contractions similar in response levels as Control animals, although now when stimulating mid-to-upper lumbar roots/cord segments, matching the spinal cord origin of obturator nerves. ObNT-ScNT Reinn animals also showed more retrograde labeling from the bladder and urethra to mid-to-upper lumbar segments of the spinal cord, further supporting the successful regrowth of axons from the lumbar cord through the transferred nerves into these end organs. Yet, only one ObNT-ScNT Reinn animal showed a return in defecation postures and lower evoked urethral and anal sphincter contractions than expected when stimulating spinal cord segments or roots (although stimulation of transferred sciatic-to-pudendal nerve branch gave much stronger results and the retrograde tracing results support regrowth of axons from the lumbar cord to the urethra). Thus, while the ScNT strategy needs further improvement or perhaps more time for integration into motor circuits for voluntary sphincter control, our results show promising outcomes for recovery of urinary end-organ function with this two-part nerve transfer strategy even after long-term denervation.

The absence of voluntarily voiding following awake cytometry testing could be a consequence of the short testing period in small cages, insufficient training, or increased anxiety during this testing procedure versus the longer video tracking of animals in their home cage that was used to generate the squat-and-void behavioral data. However, we did not observe significant recovery of defecation behaviors in nerve transfer animals (only one showed defecation posturing in their home cage in the last month; Fig. 2*B*). Yet, we observed strong anal sphincter responses when electrically stimulating the transferred sciatic-to-pudendal nerve branches during the final surgical testing (Fig. 5*C*). The latter result suggests functional reconnection of the anal sphincter across the nerve transfer site but weak brain inputs to the new sciatic-to-pudendal pathway that would drive defecation postures. In the future, one could utilize integrated neuromodulation techniques for more precise stimulation from the transferred nerve with optimize regrowth (36–38).

Surgical methods for restoring urinary function after spinal injury have been under investigation for over 100 years (14). Homotopic root repair, in which S2 and S3 roots are reconnected after transection, has been shown to result in recovery of the micturition reflex in pigs (39, 40), although root repair can be hindered by scar tissue formation if the repair procedure is delayed (41). Restoration of voluntary voiding after peripheral nerve transfer to sacral ventral roots (i.e., extradural to intradural transfer) has been shown to improve voluntary initiation of voiding in a small number of human patients, although limitations arose including sphincter dyssynergia, paresthesia in the groin and scrotum, the need for nerve grafts, and/or the need to use Valsalva straining to empty the bladder (42–44). Transfer of somatic peripheral nerves directly to pelvic nerve branches innervating the bladder has been used by our group and others to avoid sphincter dyssynergia since the sphincters are innervated by the pudendal nerve. In rats, transfer of the obturator nerve to pelvic splanchnic nerves transected 12 wk earlier resulted in the recovery of urinary function close to normal levels in the subgroup of rats that survived urinary tract infections (45). Zhang et al. (46) have published surgical procedures for rerouting peripheral nerves to the pudendal nerve, although no histological or functional outcome data was reported in support of regrowth or recovery. Our group has explored the transfer of a variety of somatic peripheral nerves to the vesical branch of the pelvic nerve or the pudendal nerve in dogs and have shown strong evoked contraction results for the bladder and sphincters, as discussed in the next two paragraphs. However, except for our pilot study (22), voluntary functional recovery was not examined until now—making this a key landmark study for our group.

Regarding the evoked contraction results, the highest evoked bladder pressures were obtained in Control animals, whether stimulating sacral spinal cord/roots or intact pelvic nerves (Fig. 3, *A*–*C*). The next highest evoked bladder pressures observed were in the ObNT-ScNT Reinn animals when stimulating the upper lumbar spinal cord or spinal roots (Fig. 3, *A* and *B*) versus the transferred obturator-to-pelvic nerve (Fig. 3*C*). Our prior study examining histomorphometric changes of the nerve coaptation site revealed significant extraneural fibrosis (i.e., scar tissue) (25). In that study, both peripheral nerve and bladder fibrosis correlated strongly with reduced evoked contractility in the bladder. Such scar tissue not only serves as an impedance of electrical stimulation but also prevents us from easily accessing the nerves for peripheral nerve stimulation without damaging the nerve in question. Perhaps the use of an antifibrotic treatment is warranted in patients with long-term spinal cord decentralization or peritoneal surgeries before surgical intervention (47, 48). However, the few anti-fibrotic pharmacotherapeutic options available (49–54) include local collagenase injections for enzymatic degradation of contractures, local corticosteroids injections to reduce inflammatory processes (both difficult for intra-abdominal scar tissue), and a drug that targets connective tissue growth factor (Pamrevlumab, a fully humanized monoclonal antibody against CCN2 (FibroGen, Inc., San Francisco, CA) that has been shown to reduce peripheral nerve fibrosis in animal models (55), yet that is not yet approved for treating peripheral nerve fibrosis in humans.

In contrast, the highest urethral and anal sphincter pressures were seen in ObNT-ScNT Reinn animals when stimulating the transferred sciatic-to-pudendal nerves rather than the spinal cord or roots (Figs. 4 and 5). We observed varying amounts of sprouting of nerves from the ventral side of the spinal cords of Decentralized and ObNT-ScNT Reinn animals at the time of tissue collection (Fig. A2). We postulate that these are motor sprouts based on their ventral location (ventral to the spinal cord and descending caudally to sacral foramina) and the increase in retrogradely labeled neurons in the mid-to-upper lumbar ventral horns, as discussed in the next paragraph. Such sprouts might not be stimulated during the spinal root segmental stimulations due to their deep ventral location, although these sprouts might be the reason for the increase in MASP in Decentralized animals when stimulating mid-to-upper lumbar root/cord segments (Fig. 4*B*). Axonal sprouting is known to occur within the spinal cord (56) as well as from avulsed ventral roots (57, 58), although they do not always improve functional recovery in adults due to a lack of directional growth. Since we were not aware of these sprouts until the time of tissue collection, we did not stimulate them. Therefore, it is unknown if they contributed to the MUSP and MASP outcomes when stimulating the sciatic-to-pudendal coapted nerve.

We used retrograde dye labeling to examine the ability of lumbar-originating nerves (obturator and sciatic nerve branches) to grow into urinary end organs. Injection of Fluoro-Gold into the bladder showed a clear and significant increase in retrogradely labeled neurons from the bladder in upper lumbar cord segments in ObNT-ScNT Reinn animals rather than in sacral segments as seen in Control animals (Figs. 6*A* and 7*A*). Injection of True Blue into the urethra showed a lower yet still significant enhancement of retrogradely labeled neurons from the urethra in upper lumbar cord segments in ObNT-ScNT Reinn animals (Figs. 6*B* and 7*B*). Thus, lumbar-originating nerves can regrow into urinary end organs (bladder and urethra in this study). Any potential input from the hypogastric nerve was eliminated in the reinnervated animals by the extensive decentralization that included hypogastric nerve transection. Interestingly, input to the bladder from L2 spinal cord segments was visible in Control animals (Fig. 6*A*). We have reported previously that stimulation of L2 roots evokes increases in bladder pressure (32). Although not included in anatomical texts, studies as far back as 1895 (59, 60) report evidence of direct inputs from thoracic, lumbar, and sacral segments in cats, humans, and dogs (30, 61). Perhaps the ease by which lumbar-originating nerves can be used to functionally reinnervate the bladder is that some circuitry already exists in the lumbar region for this purpose in at least canines.

The laminar location of the labeled nerves also shows a change with this reinnervation strategy compared with the other two groups (Fig. 8). The obturator nerve is a somatic motor nerve that has expected neuronal locations in lamina IX (this lamina is the origin of neurons that exit the ventral root to innervate appendicular muscles) of L3-L6 spinal cord segments (20, 27–29, 62, 63), while the bladder is innervated by autonomic nerves with expected neuronal locations in laminae VII (since this lamina contains neurons innervating autonomic structures) of approximately L7-S3 segments (20, 62, 64, 65). Lamina VIII contains alpha and gamma motor neurons that exit the ventral root to innervate axial muscles such as perineal and sphincter muscles) (64–66). The location of labeled neurons primarily in lamina VII in L7-S3 segments of Controls in which autonomic neurons are innervating the bladder and in lamina IX in L2-L6 segments of ObNT-ScNT Reinn animals after transfer of the obturator matches these expectations (the latter since we moved somatic nerves that innervate skeletal muscles typically). However, the ObNT-ScNT Reinn animals also showed an increased presence of labeled neurons after bladder injections of the dye in lamina VII and VIII of L2 through L6 spinal cord segments. This finding is suggestive of neuroplasticity of presumedly autonomic neurons (likely from sympathetic neurons that innervated blood vessels and erector pilae muscles in the hindlimb regions which receive obturator nerve inputs) (67). The observed sprouting of nerves from the ventral cord (Fig. A2) that descended caudally to sacral foramina at the time of euthanasia and tissue collection at 18 mo after removal of sacral ventral roots is also an indicator of peripheral nerve neuroplasticity.

To determine if the outcomes were linked and if the responsiveness of one outcome was linked to responsiveness to another outcome, we performed several correlations. Total time in study or recovery times before or after reinnervation did not affect the various outcomes (Fig. A3). Yet, there were significant correlations between squat-and-void behaviors and MDP responses after stimulation of L2-L6 spinal roots (Fig. 9*B*) and Fluoro-Gold-labeled neurons in these same upper lumbar segments (Fig. 9, *E*, *G*, and *H*). Importantly, there were correlations between MDP evoked responses and retrogradely labeled cells in L2-L6 segments (Fig. 10, *A* and *D*) that support a return of bladder function as a result of the obturator nerve transfer (which originates in L3-L6 segments in dogs (20, 27–29, 62, 63). This is particularly true for the association between MDP-evoked responses and the location of Fluoro-Gold-labeled cells in laminae IX of L2-L6 segments (Fig. 10, *A* and *D*), which indicates that somatic motor neuronal axons that originally innervated appendicular muscles now innervate the bladder. In further support of this, neuronal input from lamina IX to the bladder is missing from the Control animal correlations (Fig. 10*C*). Combined, these correlations support our hypothesis that a new neuronal pathway was achieved by transfer of the obturator nerve to vesical branches of the pelvic nerve.

There were several significant correlations between squat-and-void and defecation postures/day with MDP and MASP responses after L7-S3 spinal root stimulation (Fig. 9, *C* and *D*) and with numbers of Fluoro-Gold-labeled neurons in ventral horns of L7-S3 segments (Fig. 9, *E* and *F*). There were also correlations between MDP responses and numbers of Fluoro-Gold-labeled neurons in the ventral horn regions of L7-S3 segments in Control animals specifically (Fig. 10*C*), between MDP responses after pelvic nerve/obturator nerve stimulation and L7-S3 root stimulation (Fig. A6*A*), and between MDP and MUSP responses after L7-S3 spinal root stimulation (Fig. A6*C*). These were expected correlations since motor nerves innervating both the bladder and urethral sphincter arise from these spinal cord segments. No significant correlations were found between MDP and MUSP responses after L2-L6 spinal root versus L7-S3 spinal root stimulations (Fig. A6, *D* and *E*), as expected since upper lumbar and sacral roots give rise to different motor nerves that travel to different end organs.

This study has several limitations. We examined only female mixed-breed hound dogs to reduce variability. Inclusion of males would have entailed an additional abdominal surgery to inject retrograde dye into the bladder wall (and therefore a different dye injection method) since male dogs have a curved os penis that would have induced trauma with the use of our inflexible cystoscope instrumentation used for these injections in female dogs. The number of retrogradely labeled neurons is likely underestimated by our focal injection methods (e.g., around the ureteral orifices only in the bladder wall) and spinal cord sampling method (cross-sectional rather than longitudinal sectional examination of spinal cord segments). With these focal injections, we likely missed labeling several axon terminals in each end organ, especially for the bladder due to its large size. We chose the cross-sectional spinal cord sampling method rather than the longitudinal sectional examination of the spinal cords to examine the laminar location of labeled neurons. Underestimated or not, the significant increases in retrogradely labeled neurons in upper lumbar spinal cord segments known to give rise to the coapted obturator nerve supports a conclusion of regrowth of at least some axons from these spinal cord segments to the bladder wall. Our study was not designed to show which specific nerves carried the efferents distally; only the location of ventral horn neurons retrogradely labeled from the end organs was examined. However, the extensive decentralization, in which hypogastric nerves and sacral roots determined electrophysiologically to induce end organ contraction were transected, reduces this limitation considerably. Finally, we have yet to determine if the new neuronal pathways created by somatic nerve transfer can restore bladder sensation in long-term bladder, urethra, and anal sphincter decentralized dogs. That work is underway for Part 5 of this series, as this data is beyond the scope of this current study focused on motor behavior, evoked contractions, and regrowth of motor neurons from the spinal cord to these urinary end organs.

### Perspectives and Significance

In conclusion, while the ScNT strategy needs further improvement, our results show promising outcomes for recovery of bladder function with the ObNT strategy even after long-term denervation. Yet, even the ScNT strategy resulted in urethral and anal sphincter contractions during in vivo electrical stimulation of the urethra and anal sphincters. These results suggest the new neuronal pathways created by somatic nerve transfer can restore motor function in long-term bladder-decentralized dogs.

## DATA AVAILABILITY

Source data for this study is openly available at: http://dx.doi.org/10.34944/dspace/9955.

## SUPPLEMENTAL DATA

10.34944/dspace/9955 Videos associated with Appendix Fig. A1: http://dx.doi.org/10.34944/dspace/9955.

## GRANTS

This study was supported by National Institute of Neurological Disorders and Stroke Grant NS070267 (to M.F.B.).

## DISCLOSURES

No conflicts of interest, financial or otherwise, are declared by the authors.

## AUTHOR CONTRIBUTIONS

J.M.B., M.R.R., and M.F.B. conceived and designed research; E.T., D.S.P., A.S.B., L.H.-B,, N.A.F., J.M.B., B.R.J., S.F.B., B.A.H., M.M., M.A.P., M.R.R., and M.F.B. performed experiments; E.T., D.S.P., A.S.B., M.M., D.Y., M.R.R., and M.F.B. analyzed data; E.T., M.A.P., D.Y., M.R.R., and M.F.B. interpreted results of experiments; E.T. and M.F.B. prepared figures; E.T. and M.F.B. drafted manuscript; E.T., L.H.-B., N.A.F., B.R.J., M.A.P., D.Y., M.R.R., and M.F.B. edited and revised manuscript; E.T., D.S.P., A.S.B., L.H.-B., N.A.F., J.M.B., B.R.J., S.F.B., B.A.H., M.M., M.A.P., D.Y., M.R.R., and M.F.B. approved final version of manuscript.

## APPENDIX

Table A1 and Figs. A1–A7.

**Table A1.** Statistical findings from the mixed-effects models with statistical results given for each analysis factor

	Surgical Group	Time Point	Interaction of the Previous Two Factors	
*Behavioral outcomes*				
Squat-and-void postures	*P* < 0.0001	*P* = 0.004*	*P* = 0.001*	
Defecation postures	*P* < 0.0001	*P* = 0.0004*	*P* < 0.0001*	
*Spinal roots/cord-evoked contractions*				
	**Surgical Group**	**Individual Segments**	**Individual Segments Interaction of the Previous Two Factors**	
MDP	*P* = 0.06	*P* = 0.006*	*P* < 0.0001*	
MUSP	*P* = 0.21	*P* = 0.24	*P* = 0.003*	
MASP	*P* < 0.0001*	*P* = 0.01*	*P* < 0.0001*	
	**Surgical Group**	**Segmental Grouping**	**Interaction of the Previous Two Factors**	
MDP	*P* = 0.001*	*P* = 0.97	*P* = 0.001*	
MUSP	*P* = 0.30	*P* = 0.09	*P* = 0.004	
MASP	*P* < 0.0001*	*P* = 0.0009*	*P* < 0.0001*	
*Number of retrogradely labeled neurons in spinal cord ventral horns*				
Two-way comparisons	**Surgical Group**	**Individual Segments**	**Interaction of the Previous Two Factors**	
Neurons to detrusor	*P* < 0.0001*	*P* < 0.0001*	*P* < 0.0001*	
Neurons to urethra	*P* = 0.36	*P* < 0.0001*	*P* < 0.0001*	
Two-way comparisons	**Surgical Group**	**Segmental Grouping**	**Interaction of the Previous Two Factors**	
Neurons to detrusor	*P* = 0.14	*P* = 0.34	*P* = 0.0002	
Neurons to urethra	*P* < 0.0001*	*P* < 0.0001*	*P* < 0.0001*	
Three-way comparisons	**Surgical Group**	**Segmental Grouping**	**Lamina Location**	Interactions (If Significant)
Detrusor	*P* < 0.0001	*P* < 0.0001	*P* < 0.0001	Group × segment, *P* < 0.0001; Group × lamina, *P* < 0.0001; Group × segment × lamina, *P* < 0.0001
Urethra	*P* = 0.08	*P* = 0.69	*P* = 0.12	Group × segment interaction, *P* = 0.005

“Group” in interactions also refers to “Surgical Group;” MDP, maximum detrusor pressure; MUSP, maximum urethral sphincter pressure; MASP, maximum anal sphincter pressure.* Statistically significant.
